# Predicting the distribution of plant associations under climate change: A case study on *Larix gmelinii* in China

**DOI:** 10.1002/ece3.9374

**Published:** 2022-10-17

**Authors:** Chen Chen, Xi‐juan Zhang, Ji‐zhong Wan, Fei‐fei Gao, Shu‐sheng Yuan, Tian‐tian Sun, Zhen‐dong Ni, Jing‐hua Yu

**Affiliations:** ^1^ Institute of Applied Ecology Chinese Academy of Sciences Shenyang China; ^2^ University of Chinese Academy of Sciences Beijing China; ^3^ State Key Laboratory of Plateau Ecology and Agriculture Qinghai University Xining China

**Keywords:** climate change, *Larix gmelinii* associations, Maxent, spatial distribution, temperature

## Abstract

Association is the basic unit of plant community classification. Exploring the distribution of plant associations can help improve our understanding of biodiversity conservation. Different associations depend on different habitats and studying the association level is important for ecological restoration, regional ecological protection, regulating the ecological balance, and maintaining biodiversity. However, previous studies have only focused on suitable distribution areas for species and not on the distribution of plant associations. *Larix gmelinii* is a sensitive and abundant species that occurs along the southern margin of the Eurasian boreal forests, and its distribution is closely related to permafrost. In this study, 420 original plots of *L. gmelinii* forests were investigated. We used a Maxent model and the ArcGIS software to project the potential geographical distribution of *L. gmelinii* associations in the future (by 2050 and 2070) according to the climate scenarios RCP 2.6, RCP 4.5, and RCP 8.5. We used the multi‐classification logistic regression analysis method to obtain the response of the suitable area change for the *L. gmelinii* alliance and associations to climate change under different climate scenarios. Results revealed that temperature is the most crucial factor affecting the distribution of *L. gmelinii* forests and most of its associations under different climate scenarios. Suitable areas for each association type are shrinking by varying degrees, especially due to habitat loss at high altitudes in special terrains. Different *L. gmelinii* associations should have different management measures based on the site conditions, composition structure, growth, development, and renewal succession trends. Subsequent research should consider data on biological factors to obtain more accurate prediction results.

## Graphical abstract

Although exploring the distribution of plant associations can help improve the understanding of biodiversity conservation, the studies conducted so far have only focused on the suitable distribution areas of species and not on the distribution of plant associations. This study sought to understand the current and future changes in the distribution of *Larix gmelinii* associations to provide a scientific basis and useful reference for management, biodiversity protection, and medium and long‐term regional ecological planning.

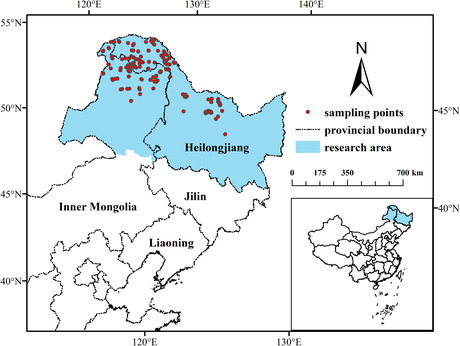

## 1 INTRODUCTION

The continuous emission of greenhouse gases is now widely credited for causing global warming (Allen et al., 2010; Friend et al., 2014; Kamkeng et al., 2021; Meinshausen et al., 2009). Generally, climate change has far‐reaching impacts on species ranges, leading to changes in species dominance, survival, succession, and community structure (Crase et al., 2015; Fei et al., 2017; Pires et al., 2018). Typically, forests play an important role in the global carbon cycle (Bonan, 2008; Pan et al., 2011; Schlosser et al., 2003), and the dominant effect of climate change on forest ecosystems is evident at low and high altitudes (He et al., 2005). The Chinese boreal forests are on the southern margin of the Eurasian boreal forests (Jia et al., 2021). *Larix gmelinii* is commonly found in the boreal forests of subalpine coniferous forests in Northeast China and contributes to the forests' high carbon storage capacity (Fang et al., 2001; He et al., 2019). The range of *L. gmelinii* extends almost to the permafrost region (Larionova et al., 2004). A particular concern is that the northern boundary of the broad‐leaved forest is moving northwest (Chen, 2000). Li et al. (2006) found that the geographical distribution of *L. gmelinii* forests is decreasing and may even move northward from China. Yang et al. (2014) indicated that suitable high‐altitude areas for larch forests are not available in China.

Dominant species (especially constructive species) coexist with the community, are important builders of the community and create a specific community environment (Zhou, 1991). In this paper, the *L. gmelinii* associations of different dominant shrub and grass species were taken as the research object. Biodiversity is indispensable for stabilizing biological communities (Loreau & de Mazancourt, 2013; Ma et al., 2017; Mougi & Kondoh, 2012). Species in ecological communities reflect the interactions among organisms and between organisms and their abiotic environments (Cardinaux et al., 2018; Koffel et al., 2021; Walther et al., 2002). Many researchers have focused on the response of communities to global changes, and an in‐depth understanding of species interactions can help to predict their responses to climate change (Enquist, 2002; Gilman et al., 2010; Ovaskainen et al., 2013; Santos‐Hernández et al., 2021). Climate change can lead to inconsistencies in the phenology of species, which in turn leads to community changes (Ovaskainen et al., 2013). Through long‐term observations, it has been found that with climate change, cold mountain habitats and the biological communities in high mountains are gradually decreasing (Gottfried et al., 2012). Therefore, conserving habitats and maintaining the living conditions of this species is vital, given that larch habitats support a wide range of organisms, including endemic species, and that any habitat change can affect their distribution (Rivas et al., 2020).

The Chinese vegetation classification system is separated into three levels, namely vegetation, alliance, and association (Fang & Wang, 2020; Wang et al., 2020), with the association being the basic unit of plant community classification (Jennings et al., 2009; Tansley, 1920). This study addressed the following research questions: (1) Which climatic factors have the power to distribute the *L. gmelinii* associations more strongly? (2) Which association types control the movement of *L. gmelinii* forests under different climate change scenarios?

Compared to field surveys, the study of plant communities using remote sensing methods does not provide sufficiently comprehensive results. For example, the spectral signal changes between communities are not evident when using remote sensing, and the ability to interpret complex local terrains is limited (Chang et al., 2004; Westman et al., 1989).In this study, 420 original plots of *L. gmelinii* forests were investigated. The forest plot area was set to 30 × 30 m, and the sample plot survey data included the basic condition of the tree, shrub, and herb species in the plot. The Maxent model and the ArcGIS software can help determine the future (by 2050 and 2070) potential geographical distribution of different associations based on the three different climate scenarios of RCP 2.6, RCP 4.5, and RCP 8.5 (Dyderski et al., 2018; Tapiador et al., 2019).

The reasons for the changes in spatial distribution can be analyzed using multinomial logistic regression analysis (Fagerland et al., 2008; Friedman et al., 2010; Kwak & Alan, 2002). Through this study, we seek to understand the current and future changes in the distribution of *L. gmelinii* associations to provide a scientific basis and useful reference for medium and long‐term management, biodiversity protection, and regional ecological planning.

## 2 MATERIALS AND METHODS

### 2.1 Study area

**FIGURE 1:**
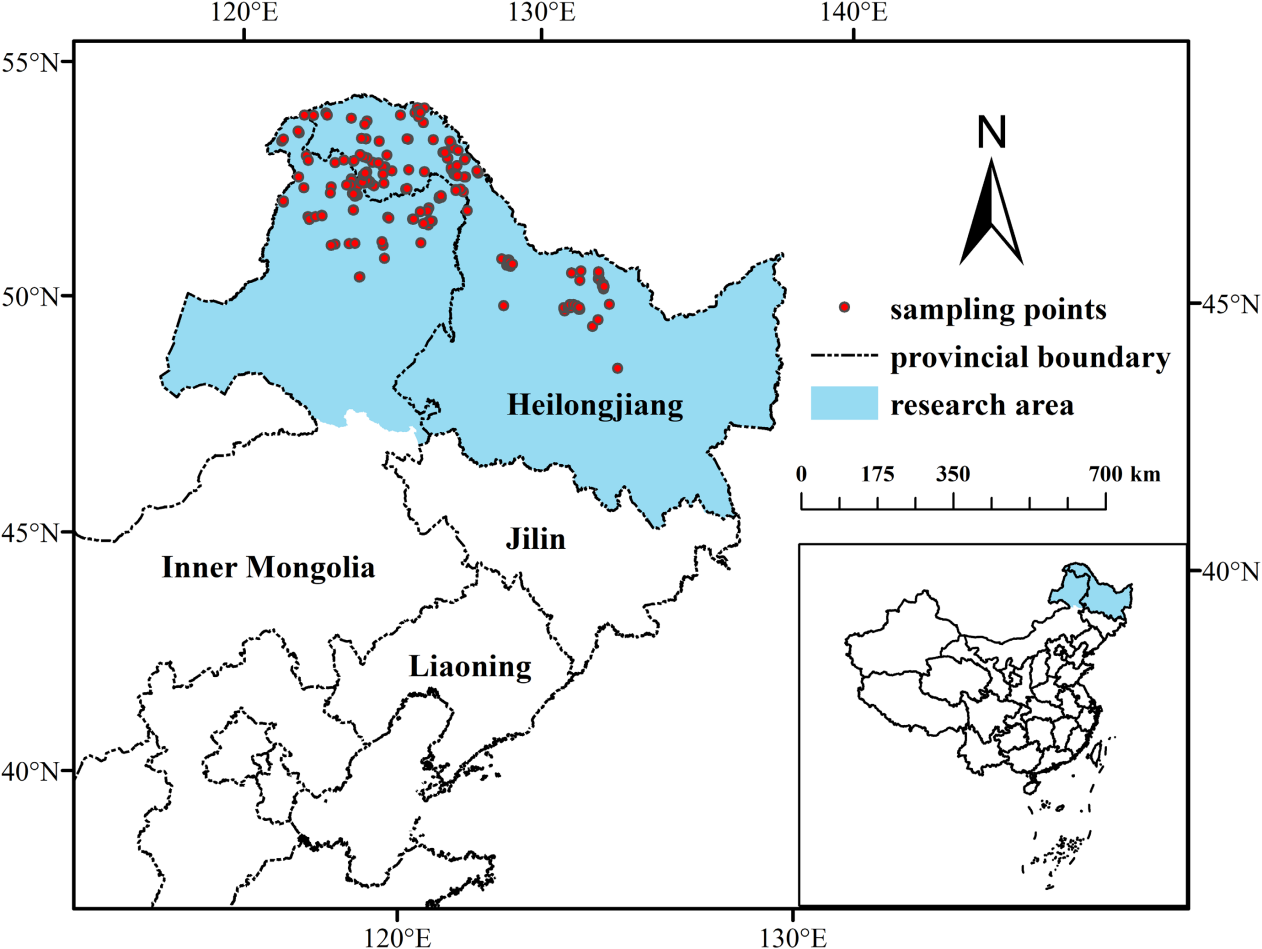
The geographical location of the research area. The blue part represents the research area, the red points represent the sampling points, and the black lines represent the provincial boundaries.

The study area is located in Northeast China, with a geographical range of 43°25′N–53°33′N and 115°31′E–135°05′E encompassing an area of 0.723 million km^2^. The northern part of the Greater Khingan Mountains is the only high‐latitude cold temperate region and is the second largest permafrost region in China (Duan et al., 2017, 2020). (Figure 1).

### 2.2 Data analysis

#### 2.2.1 Sample plot data

Based on regional distribution data from Northeast China, 420 plots of *L. gmelinii* forests were selected for this study. Zhou (1991) divided the association based on the same layer structure; the dominant species or co‐dominant species of each layer are the same plant community. The data were classified using two‐way indicator species analysis (Hill et al., 1975) combined with traditional community classification (Zhou, 1991, 1994, 1997) to remove transitional associations and were assigned names. For example, Ass. *Carex callitrichos*, *Rhododendron davuricum*, *Larix gmelinii* (LRC1) and Ass. *Vaccinium vitisidea*, *Rhododendron davuricum*, *Larix gmelinii* (LRV3) have the same association group but LRC1 is the association where *C. callitrichos* is the dominant species, and LRV3 is where *Vaccinium vitisidea* is the dominant species. We set a buffer radius of 1 km to screen the distribution points of the plots to avoid the influence of overfitting caused by excessive correlation. Subsequently, 13 association types were determined (Table 1). We then processed all the association distribution points with a 1 km buffer to obtain the points at the level of the *L. gmelinii* alliance, with 182 distribution points for the *L. gmelinii* alliance being available.

**TABLE 1:** Association type

Code	Association^a^	Quantity	Category
LRC1	Ass. *Carex callitrichos*, *Rhododendron davuricum*, *Larix gmelinii*	15	Mesogenic drought
LRD2	Ass. *Deyeuxia pyramidalis*, *Rhododendron davuricum*, *Larix gmelinii*	6	Mesogenic drought
LRV3	Ass. *Vaccinium vitisidea*, *Rhododendron davuricum*, *Larix gmelinii*	20	Mesogenic drought
LLC4	Ass. *Carex callitrichos*, *Lespedeza bicolor*, *Larix gmelinii*	7	Mesogenic drought
LH5	Ass. *Herbage*, *Larix gmelinii*	54	Mesogenic
LCC6	Ass. *Carex lanceolata*, *Corylus mandshurica*, *Larix gmelinii*	40	Mesogenic
LCC7	Ass. *Carex callitrichos*, *Corylus heterophylla*, *Larix gmelinii*	20	Mesogenic
LPV8	Ass. *Vaccinium vitis‐idaea*, *Pinus pumila*, *Larix gmelinii*	15	Mesogenic
LLV9	Ass. *Vaccinium vitis‐idaea*, *Ledum palustre*, *Larix gmelinii*	38	Mesogenic wet
LV10	Ass. *Vaccinium vitis‐idaea*, *Larix gmelinii*	8	Mesogenic wet
LBC11	Ass. *Carex schmidtii*, *Betula fruticosa*, *Larix gmelinii*	42	Wet
LBV12	Ass. *Vaccinium vitis‐idaea*, *Betula fruticosa*, *Larix gmelinii*	14	Wet
LBV13	Ass. *Vaccinium vitis‐idaea*, *Betula middendorfii*, *Larix gmelinii*	5	Wet

^a^ Please refer to Appendix 1 for a detailed introduction to *Larix gmelinii* associations.

#### 2.2.2 Environmental data

The WorldClim database (http://worldclim.org) can describe climatic conditions by specifying annual and seasonal changes in temperature and precipitation. We used the “WorldClim 2” dataset at a spatial resolution of 30 arcs, commonly referred to as “1‐km” spatial resolution (Fick & Hijmans, 2017). We then separated the future period into 2050 and 2070. CMIP5 implemented four representative concentration pathways (RCP 2.6, RCP 4.5, RCP 6.0, and RCP 8.5) which describe the change curves of different greenhouse gas concentrations in response to different levels of increase in radiative forcing (IPCC, 2013). CMIP6 employed the shared socioeconomic pathways (SSPs), working in harmony with RCPs via shared policy assumptions (The CMIP6 Landscape, 2019). Our study used field survey data, which requires close‐to‐natural simulations, so the policy‐oriented CMIP6 scenario was not selected. In terms of global warming, RCP 8.5 showed the most pessimistic condition, RCP 2.6 showed the most optimistic condition, whereas RCP 4.5 showed moderate conditions. Based on previous research (Dyderski et al., 2018; Tapiador et al., 2019; Yu et al., 2019), we selected three climate change scenarios (RCP 2.6, RCP 4.5, and RCP 8.5) for prediction and analysis using Maxent.

Variables such as the soil and terrain are difficult to predict but can be regarded as static variables and input into the maxent model to obtain more accurate results (Stanton et al., 2012). The soil data were obtained from the World Soil Database at a spatial resolution of 1 km (Science Data Center for Cold and Dry Areas, Chinese Academy of Sciences, Lanzhou, China; http://westdc.westgis.ac.cn/). Topographic data were obtained at an altitudinal spatial resolution of 90 m (Resource and Environmental Science Data Center, Chinese Academy of Sciences, Beijing, China; http://www.resdc.cn/). All the environmental factors involved were unified using the coordinate system WGS1984 and were resampled to the same resolution.

To increase the accuracy of the model results, the environmental variables selected were subjected to multiple collinearity tests. First, we submitted the climate and soil variables into the Maxent model as input data for the initial operation and then calculated the contribution values of 19 climate variables, three topographic factors, and 41 soil variables. Subsequently, we used the R 4.2.0 package “ENMTools” (Warren et al., 2021) to conduct Pearson's correlation analysis. Based on the environmental contribution rate of the initial model, if the correlation coefficient of the two variables was greater than 0.8, the environmental variable with a larger contribution rate was selected, the actual distribution of species was determined, and relevant research results were examined (Yang et al., 2013, 2014). The factors such as the soil moisture content (Yang et al., 2017) and the annual mean temperature (Jia et al., 2021) with ecological significance were saved by referring to the relevant research results. Finally, six climatic variables, three topographic factors, and five soil factors were selected (Table A1 in Appendix 1 and Table 2). Environmental variables have been proven to affect the distribution and physiology of plant species across different spatial extents (from local to global scales) and are widely used to project the distributions of plant species.

**TABLE 2:** Contribution rate of major environmental factors

Variables	Description	Unit	Category
bio01	Annual Mean Temperature	°C × 10	Temperature
bio03	Isothermality (Daily average range/Temperature Annual Range) (×100)	‐	
bio04	Temperature Seasonality (Standard deviation*100)	‐	
bio12	Annual Precipitation	mm	Precipitation
bio13	Precipitation of Wettest Month	mm	
bio15	Precipitation Seasonality (Coefficient of variation)	%	
cate1	SU_SYM90 (Soil name in FAO90 soil classification system)	‐	Soil type
cate6	SWR (soil moisture content)	‐	
cont16	T_CACO3: Real (Surface carbonate or lime content)	%weight	Soil physical and chemical properties
cont30	S_BS: Real (Basic saturation of bottom layer)	%	
cont32	S_CACO3: Real (Bottom carbonate or lime content)	%weight	
Dem	Altitude	m	Terrain
Slope	Slope	°	
Aspect	Slope aspect	‐	

#### 2.2.3 Model analysis

Species distribution models (SDMs) provide comprehensive distribution statements of possible future occurrences by connecting the existence of species with condition predictors (Despland & Houle, 1997; Zhao et al., 2020; Zhong et al., 2021). Maxent shows higher performance and accuracy than other SDM tools (Carnaval & Moritz, 2008). It also has a good prediction ability for small sample datasets (Elith et al., 2011; Pearson et al., 2006; Phillips et al., 2006). It can also be used to identify areas where sensitive species currently exist or may exist (Li et al., 2020; Qin et al., 2017). The Maxent model indirectly describes how ecological processes shape ecological communities in the form of constraints (Bertram et al., 2019), and simulates the sample and environmental data of vegetation at the local and regional scale (Comino et al., 2021; Merow et al., 2013; Phillips et al., 2017; Radosavljevic & Anderson, 2014).

**FIGURE 2:**
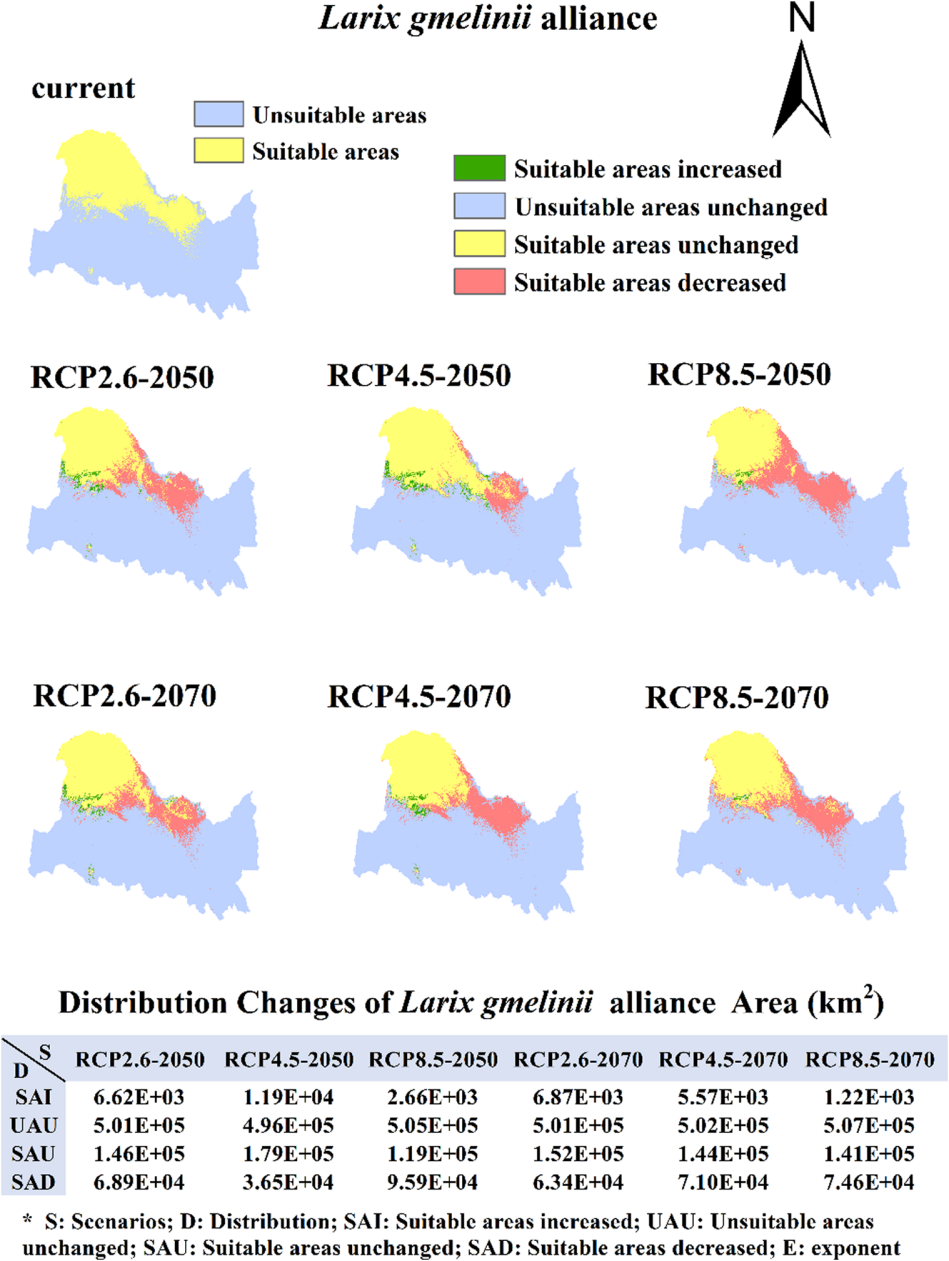
Current and potential future (2050 and 2070) geographical distribution of the *Larix gmelinii* alliance based on the climate scenarios RCP 2.6, RCP 4.5, and RCP 8.5.

**FIGURE 3:**
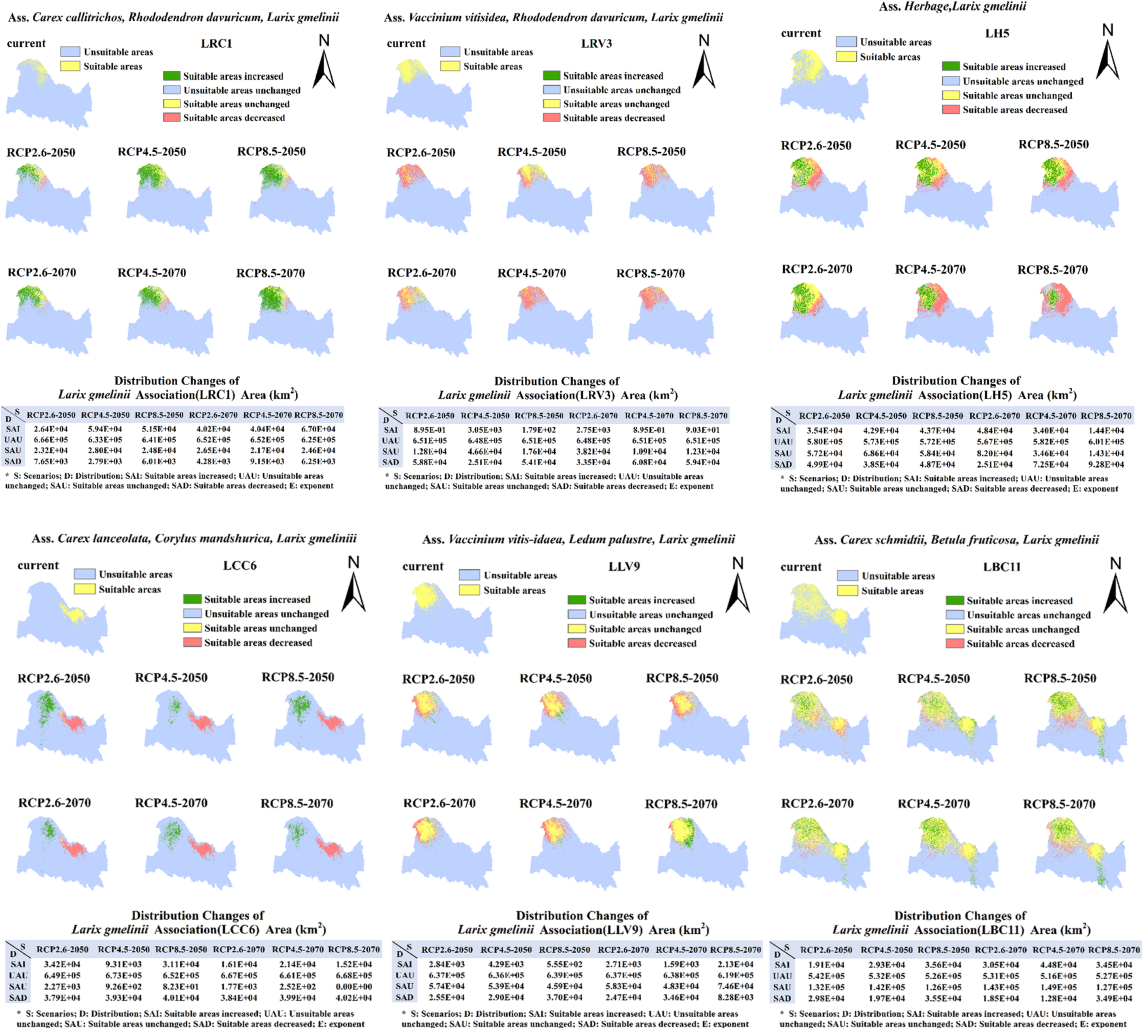
Current and potential future (2050 and 2070) the geographical distribution of *Larix gmelinii* associations based on the climate scenarios RCP 2.6, RCP 4.5, and RCP 8.5.

Considering the association as a species, we used the Maxent model to quantitatively prove its association with environmental factors and explore the response of association distribution to climate change. Given that the quantity of *L. gmelinii* associations is different, we operate the model according to the following rules (Elith et al., 2011). By default (auto features), when the sample is greater than 80, all features were used. If the quantity range of the sample was 2–9, “Linear features” was selected. If the quantity range of the sample was 10–14, “Linear features” and “Quadratic features” were selected. If the sample's quantity range was 15–79, “Linear features”, “Quadratic features,” and “Hinge features” were selected. We randomly selected 75% of the distribution data as the training set to establish a prediction model, and the remaining 25% were used as the test set for model validation (Zhang et al., 2016). The maximum number of iterations was 1000, and the number of model repetitions was 10 (Salako et al., 2019). Jackknife analysis using Maxent was performed to determine the weight of each variable (Zhang et al., 2016). The receiver operating characteristic curve analysis method was used to verify the accuracy of the Maxent model prediction results (Hanley & McNeil, 1982). In this method, the prediction accuracy of the model is determined by calculating the area under the curve (AUC) value (Swets, 1988). When the AUC value is greater than 0.9, the prediction accuracy is high, and the prediction results can be used. The Maxent model outputs the existence probability of *L. gmelinii* alliance and associations for each grid point in ASCII format, and uses ArcGIS to convert the data into Raster format to give the potential distribution map of the alliance and associations. We selected the “minimum training presence logistic threshold” (Itzel Montemayor et al., 2016), which is the 10‐time average of the maxent output, to distinguish between suitable and unsuitable regions for species and to visualize the model results. To further quantitatively analyze the changes in the spatial pattern of the *L. gmelinii* alliance and their associations, we defined four types of conditions: suitable areas increased, unsuitable areas unchanged, suitable areas unchanged, and suitable areas decreased. The SDM toolbox (Brown, 2014; http://www.sdmtoolbox.org/downloads) was used to determine the spatial pattern change of the *L. gmelinii* alliance and associations under different future climate scenarios (Figures 2 and 3). This is based on the current distribution simulated by maxent, with suitable areas as 1 and unsuitable areas as 0. The simulated distribution under the future climate scenario was compared with the current situation; range expansion (suitable areas increased) was considered as −1, no occupancy (unsuitable areas unchanged) as 0, no change (suitable areas unchanged) as 1, and range contraction (suitable areas decreased) as 2.

#### 2.2.4 Multinomial logistic regression analysis

The dependent variable consisted of disordered multi‐classification data, which were suitable for the multinomial logistic regression model (Fagerland et al., 2008; Friedman et al., 2010; Kwak & Alan, 2002). We used the multi‐classification logistic regression analysis method to obtain the response of the suitable area change for the *L. gmelinii* alliance and associations to climate change under different climate scenarios.

(1) $$\ln\left(\frac{P\left(y=j|X\right)}{P\left(y=J|X\right)}\right)=\beta_{i0}+\sum_{i=1}^{k}\beta_{\mathrm{ji}}X_{i}\;$$

where *β*_0_ is a constant, I = 1, 2, …, *k*; *j* = 1, 2, …, *J* − 1, and *X*_i_ is an explanatory variable.

We selected 182 distribution points for the *L. gmelinii* alliance in the study area, and the distribution changes and climatic factor changes at the 182 points were extracted as modeling data. Taking RCP2.6‐2050 as an example, subtracting the value of the current scenario grid from the value of the future scenario grid was the amplitude of the change. We extracted the charge values of amplitude for the environmental factors and the change in the distribution of points using the “Extract Multi Values to Points” tool in ArcGIS software. The change in the distribution of points (−1, 0, 1 and 2) was considered as the dependent variable *J* and the amplitude of the main climatic factors (bio01, bio03, bio04, bio12, bio13, bio15) as the independent variable *X*_*i*_. The climatic factors that dominated the change in the *L. gmelinii* association distribution were analyzed using a multinomial logistic regression model, which was more conducive to an in‐depth analysis of the relationship between changes in suitable areas and climatic factors. It can study which climate factors would considerably impact the increase or decrease in the suitable area and whether the correlation was negative or positive.

## 3 RESULTS

### 3.1 Current and potential future geographical distribution of *Larix gmelinii* alliance and associations

The prediction accuracy was tested, and the mean AUC value of the test dataset was greater than 0.9, which showed that the simulation accuracy of the potentially suitable area using Maxent was high, and the prediction results were reliable (Figures A3 and A4 in Appendix 1). The distribution of the *L. gmelinii* alliance and the associations based on Maxent were visualized (Figures 2 and 3). The distribution point of the *L. gmelinii* alliance into the maxent is the sum of 13 clusters which were obtained after removing the 1 km buffer. The average training AUC for the replicate runs is 0.946, and the standard deviation is 0.005. The jackknife test of variable importance showed that the environmental variable with the highest gain, when used in isolation, is bio04 (temperature seasonality), and the environmental variable that decreases the gain the most when omitted is bio12 (annual precipitation). Under the three future climate scenarios, the boundary of the suitable area for the *L. gmelinii* alliance will migrate by different degrees by 2050 and 2070. It is predicted that the southern boundary will move northward, the eastern boundary will move slightly westward, the western and northern boundaries will not substantially change, and the centroid will move northwestward. The main change in the distribution area was the decrease in the suitable area with a loss rate of 16.95%–44.58%.

**FIGURE A1:**
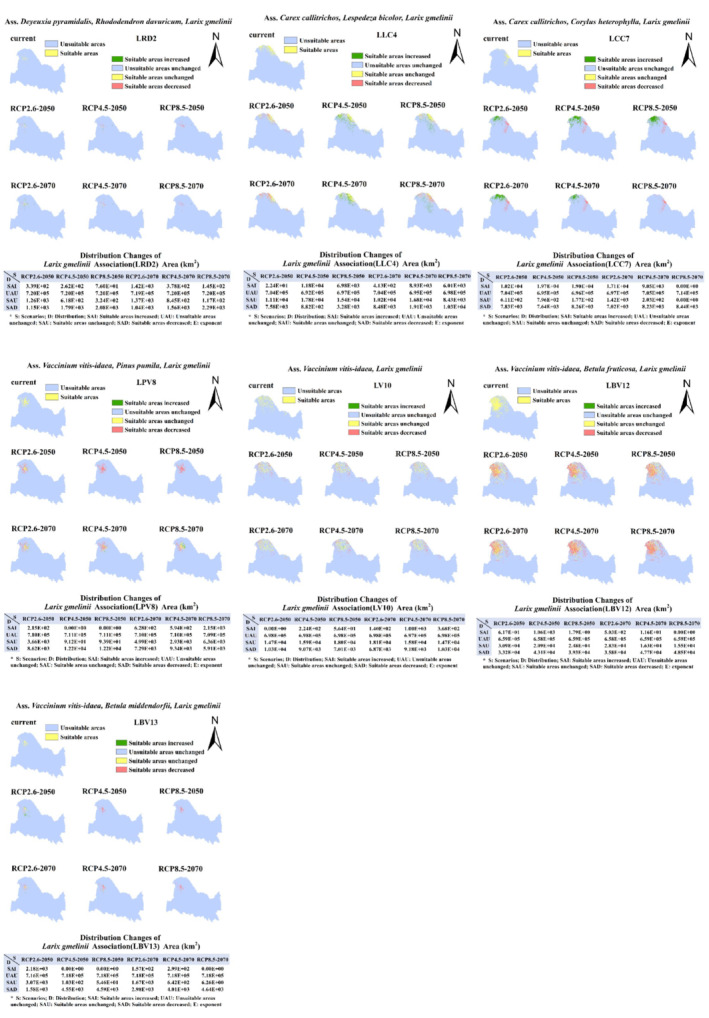
Current and potential future (2050 and 2070) geographical distribution of *Larix gmelinii* associations based on the climate scenarios RCP 2.6, RCP 4.5, and RCP 8.5

These results further highlight that the areas suitable for *L. gmelinii* associations in its future distribution will also decrease. However, there were significant differences in the distribution of different association types. Some of the *L. gmelinii* association distribution results are used as an example (the remainder of the results are in the Appendix 1, please refer to Figure A1) below. The examples selected are *L. gmelinii* associations with different habitats and additional sampling points, including LRC1 and LRV3 which are important in mesogenic drought habitats, LH5 and LCC6 for mesogenic habitats, LLV9 for mesogenic wet habitats, and LBC11 for wet habitats (Figure 3). The northern area suitable for LRC1 has considerably increased. The main distribution in the scattered areas for LRV3 will decrease further. Although the LH5 suitable habitat will decrease, it will also show a pronounced increase in the Northwest. The habitat loss rate of LCC6 will be relatively high, but this type will occupy a new northward habitat. The shrinkage of suitable areas for LLV9 and LBC11 was relatively less than that of the other types, and there was also an increase in suitable areas.

### 3.2 Importance of environmental factors in the *Larix gmelinii* alliance and associations

**FIGURE 4:**
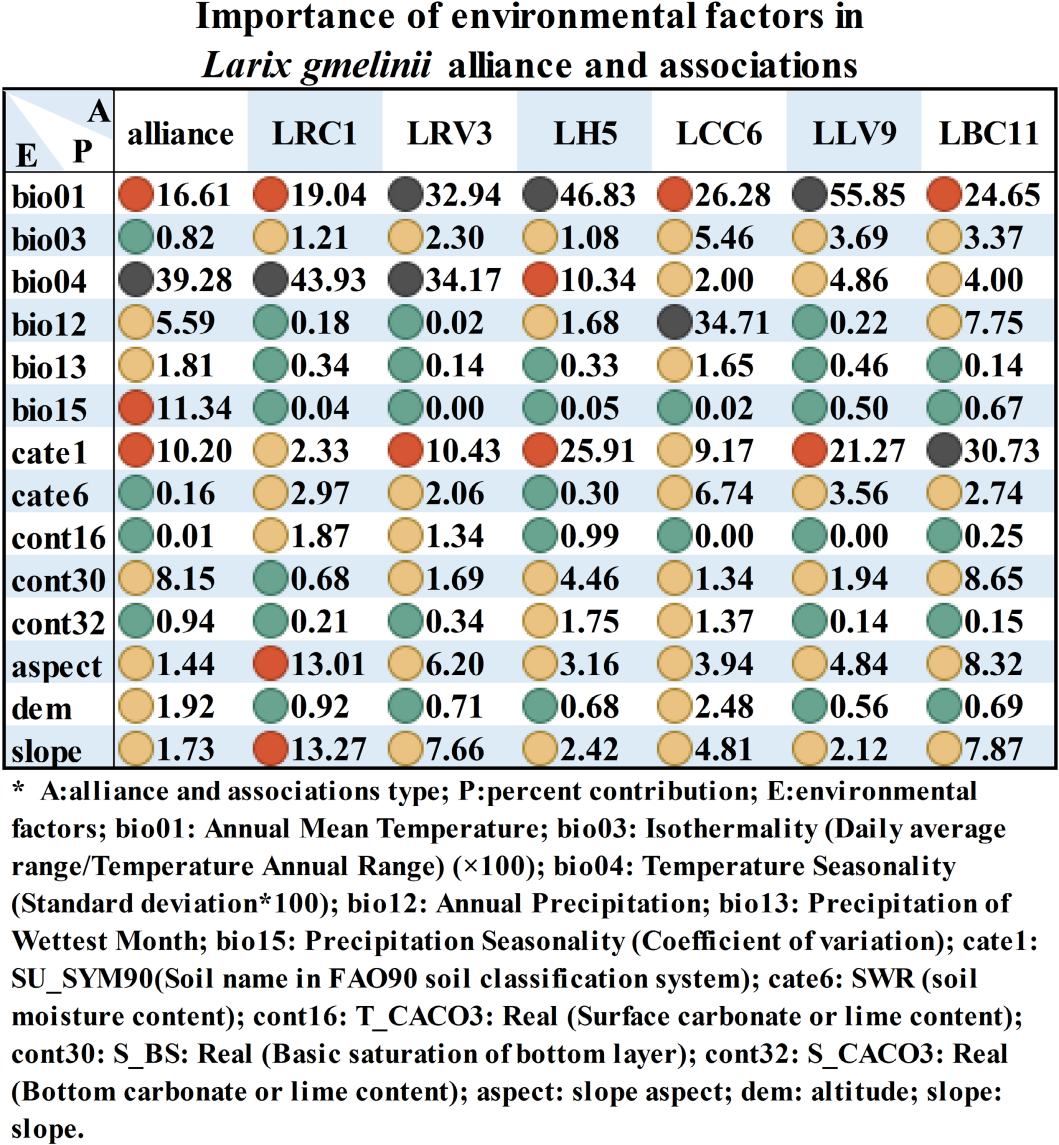
Importance of environmental factors in *Larix gmelinii* alliance and associations. The table gives estimates of relative contributions of each environmental variable to the Maxent model, and the values shown are averages over 10 replicate runs. Black circles indicate percent contribution ≥30%, red circles indicate 10% ≤ percent contribution < 30%, yellow circles indicate 1% ≤ percent contribution < 10%, green circle indicate percent contribution < 1%. Please refer to Table 2 for an explanation of the environmental factors.

**FIGURE A2:**
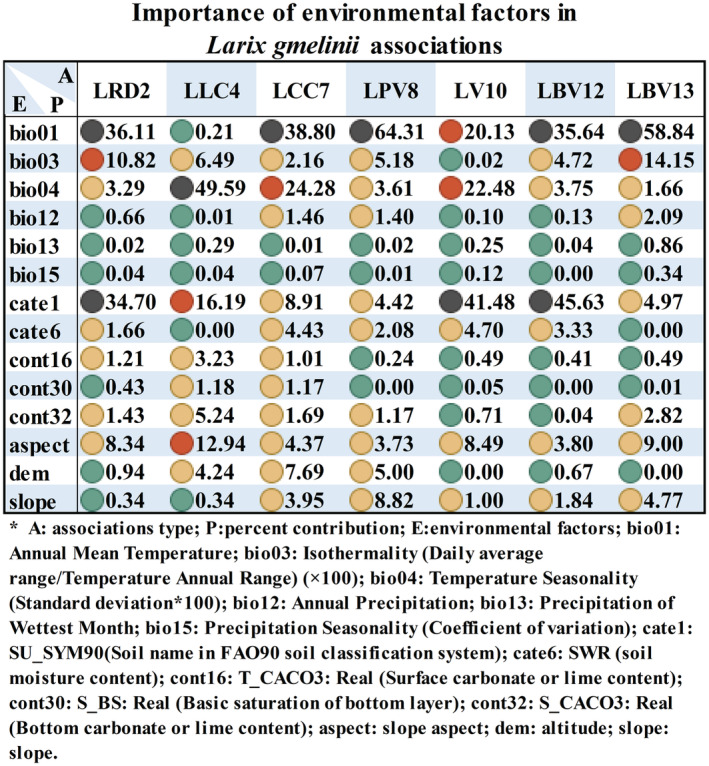
Importance of environmental factors in *Larix gmelinii* associations. The table gives estimates of relative contributions of the environmental variables to the Maxent model, and the values shown are averages over the 10 replicate runs. Black circle means percent contribution ≥30%, red circle means 10% ≤ percent contribution < 30%, yellow circle means 1% ≤ percent contribution < 10%, green circle means percent contribution < 1%. Please refer to Table 2 for an explanation of the environmental factors.

**FIGURE A3:**
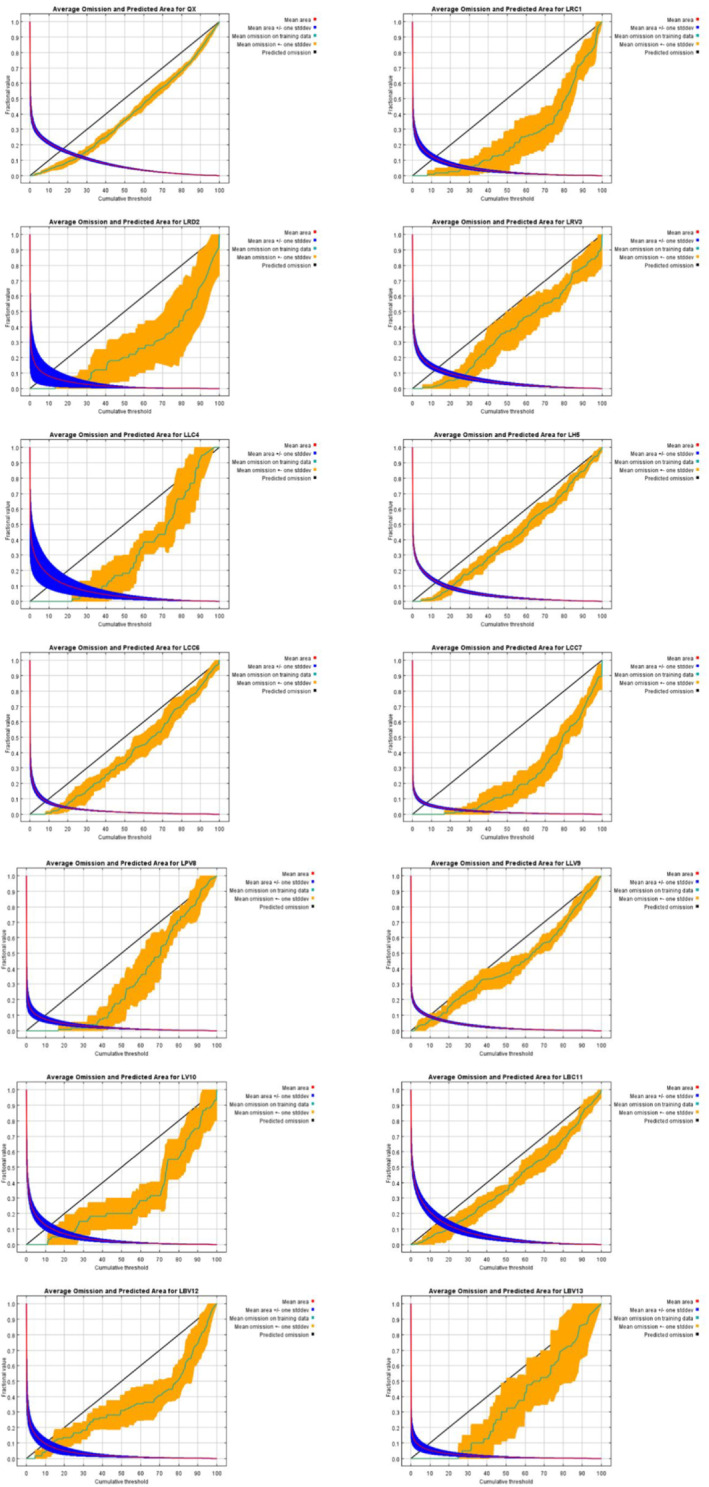
Average omission and predicted area for *Larix gmelinii* alliance (QX) and associations. The picture shows the training omission rate and predicted areas as a function of cumulative threshold, averaged over the 10 replicate runs.

**FIGURE A4:**
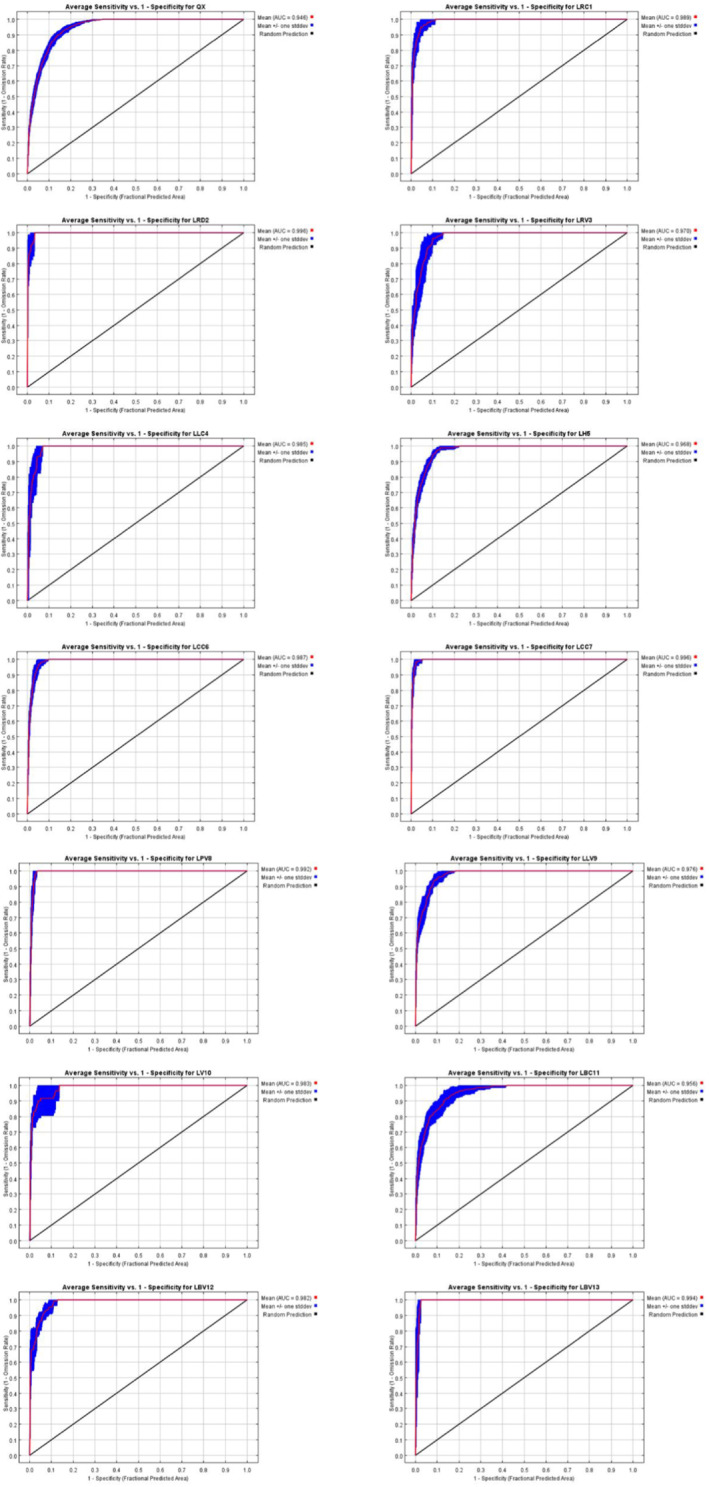
The receiver operating characteristic (ROC) curve for *Larix gmelinii* alliance (QX) and associations.

The output results of the three climate scenario models for the two periods were analyzed, and the contribution rates of each environmental factor involved in the modeling were statistically analyzed according to the jackknife method provided by the model. The statistical results for the contribution rates are shown in Figure 4 (please refer to Figure A2 in Appendix 1). The results showed that among the environmental factors assessed in the modeling, bio04 had the highest contribution rate (39.28%), indicating that temperature seasonality was the most important environmental factor affecting the distribution of the *L. gmelinii* alliance. The contribution rate of bio01 was 16.61%, while that of cate1, cont30, and bio12 were 10.20%, 8.15%, and 5.59%, respectively. The environmental factors were divided into four categories, namely temperature, precipitation, topography, and soil. The main factors affecting the distribution of *L. gmelinii* alliance were in the order temperature (56.71%), soil (19.46%), precipitation (18.74%), and terrain (5.09%). However, there were considerable differences in the factor contribution rates for each of the *L. gmelinii* associations. The main factors affecting the distribution of association LRC1 were in the order temperature (64.18%), terrain (27.19%), soil (8.06%), and precipitation (0.56%). The main factors affecting the distribution of associations LCC6 were in the order, precipitation (36.39%), temperature (33.75%), soil (18.64%), and terrain (11.23%). The dominant factors affecting the distribution of associations LRV3, LH5, LLV9, and LBC11 were in the order, temperature, soil, terrain, and precipitation. This also demonstrated that the suitable habitats for different *L. gmelinii* association types are different.

### 3.3 Response of the spatial distribution of *Larix gmelinii* alliance and associations to climate change

**FIGURE 5:**
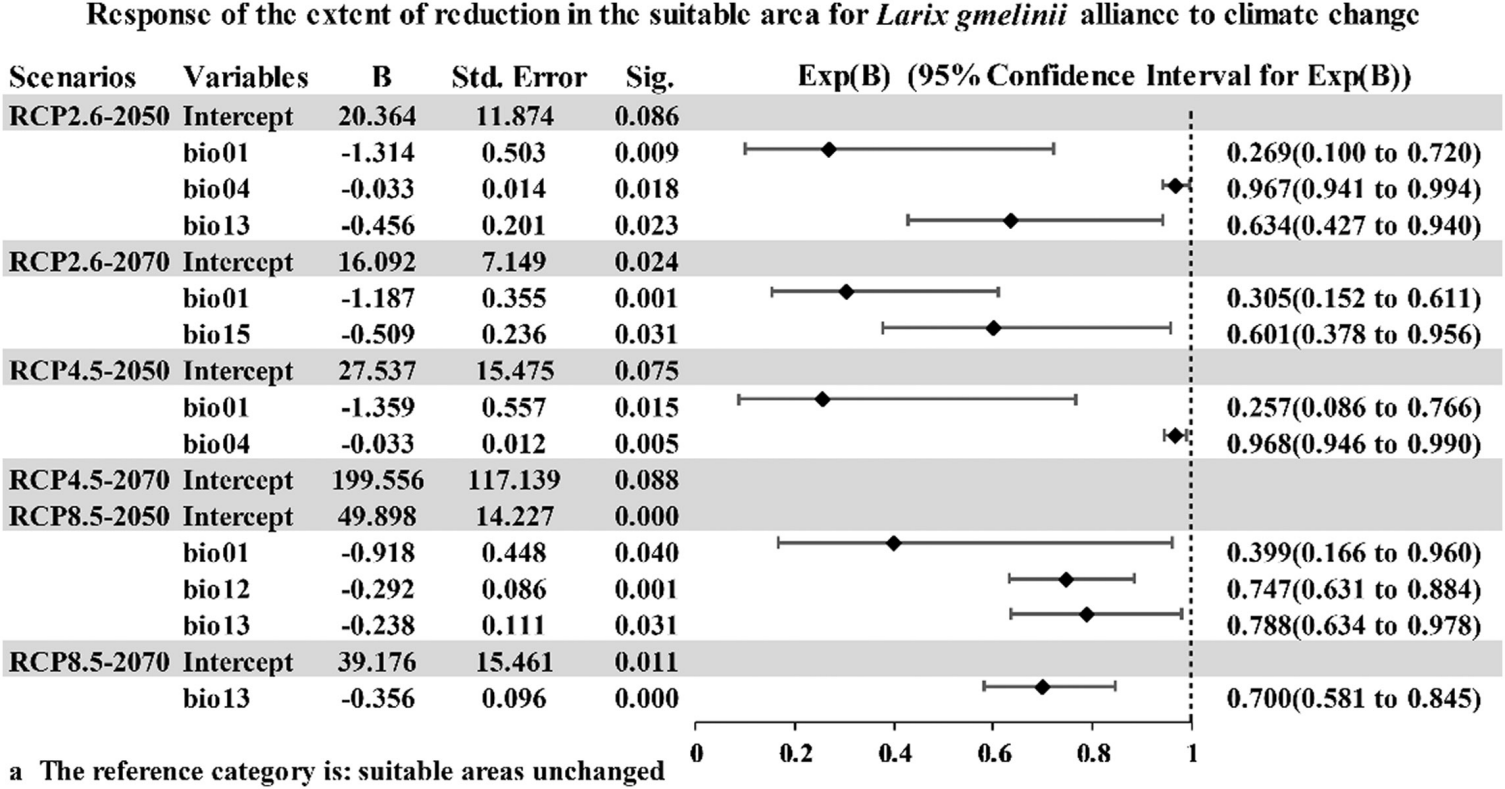
Response of the extent of reduction in the suitable area for *Larix gmelinii* alliance to climate change. B, the regression coefficient; Sig: *p*‐values; Sig < .05 is considered to be significant based on the coefficient test (numeric results rounded to three decimal places), indicating that B is meaningful. Exp(B) is the OR (odds ratio) value which is compared with 1; values closer to 1 indicate smaller degrees of influence, and vice versa.

**FIGURE 6:**
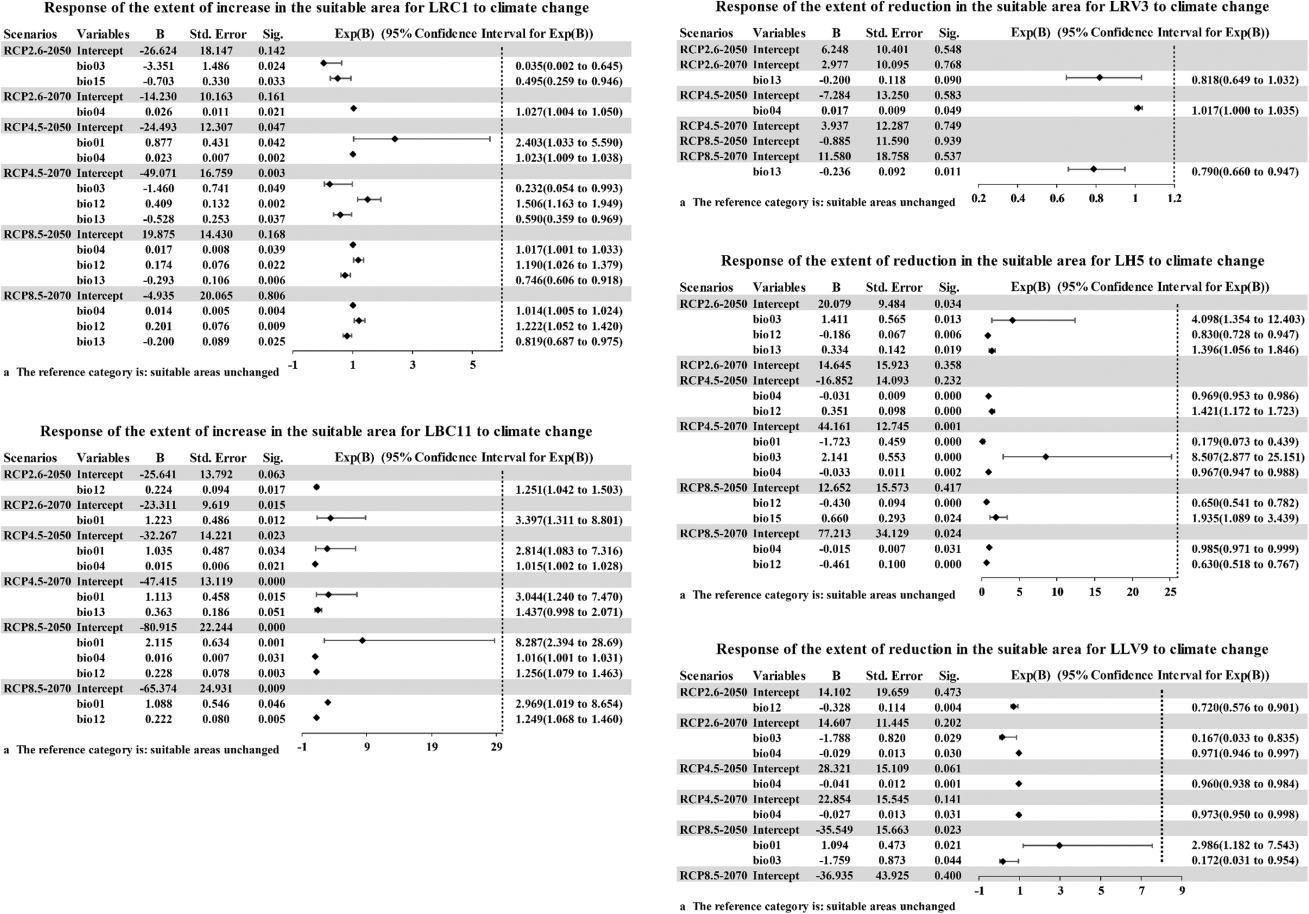
Response of the extent of increase or reduction in the suitable area for *Larix gmelinii* associations to climate change. B, the regression coefficient; Sig: *p*‐values; Sig < .05 is considered to be significant based on the coefficient test (numeric results rounded to three decimal places), indicating that B is meaningful. Exp(B) is the OR (odds ratio) value which is compared with 1; values closer to 1 indicate smaller degrees of influence, and vice versa.

In this study, we used the entire suitable area as a reference and aimed to analyze the extent of increase or reduction in the suitable area. A multinomial logistic regression model was used to determine the climatic factors that impact *L. gmelinii* forests and their spatial change associations. A significance value less than .05 indicates that the coefficient of the corresponding independent variable is statistically significant and has a significant impact on the changes of the dependent variable at different classification levels. Considering the unchanged suitable area as the reference group, the results of the model operation were statistically significant. The distribution pattern of the suitable area increasing or decreasing in the *L. gmelinii* alliance and associations under different climate scenarios was screened using various factors of the visibility test. The corresponding results are shown in Figures 5 and 6.

The response of the spatial distribution of the *L. gmelinii* alliance to climate change was analyzed as follows: For most of the scenarios, the change in the annual mean temperature (bio01) would have a considerable impact on the reduction of the suitable area, and the correlation was negative. Under the RCP2.6 scenario, temperature and precipitation would significantly contribute to habitat loss. Under the RCP4.5 scenario, by 2050, the temperature would play a key role in reducing the suitable area. Under the RCP4.5 scenario, by 2070, because the *p* value is not statistically significant, it cannot reflect the impact of climatic factors on the reduction in suitable areas. Under the RCP8.5 scenario, by 2050 and 2070, the change in the Precipitation of Wettest Month (bio13) would have the most impact on the reduction in the suitable area.

According to the current and future changes in the suitable area, the response of the extent of increase in the suitable area for LRC1 and LBC11 to climate change and the extent of reduction in the suitable area for LRV3, LH5, and LLV9 to climate change were analyzed. The unchanged suitable area for LCC6 was too small or even zero, and the results were not statistically significant, so this was not listed in the results. Compared with the contribution from the variables analyzed in the Maxent model, some variables such as precipitation with a relatively low contribution also play a role in causing an increase or reduction in the suitable area. For most of the *L. gmelinii* associations, the mean annual temperature (bio01), temperature seasonality (bio04), annual precipitation (bio12), and precipitation of the wettest month (bio13) could impact the expansion or contraction of the suitable area. Under the RCP4.5 scenario, by 2050, changes in the mean annual temperature would cause the most impact on the increase in the suitable area distribution for LRC1. Under the RCP8.5 scenario, by 2050, changes in the mean annual temperature would also cause the most impact on the increase in the suitable area distribution of LBC11 and the reduction of the suitable area distribution of LLV9. Under the RCP 4.5 scenario, by 2070, Isothermality (bio03) would have the greatest impact on LH5 on the reduction in the suitable area, and the correlation was positive.

## 4 DISCUSSION

Dominant species can alter the living conditions of other species and affect the entire community (Hickler et al., 2012). At the community scale, forests are a mixture of tree species with different functional characteristics and growth behaviors that respond to different light, moisture, and nutrient regimes (Pan et al., 2013). Due to different site conditions, the dominant species in Northeast China, *L. gmelinii*, can form different association types with other species. The *L. gmelinii* forest, as the top vegetation, is distributed under different site conditions, and the analysis accuracy from the perspective of the alliance is not enough. The associations in our study were divided into four categories based on the vegetation type, namely mesogenic drought, mesogenic, mesogenic wet, and wet association. *Rhododendron davuricum* can survive on an upper dry, sunny slopes, *Ledum palustre* grows in humid areas, and *Betula fruticosa* tends to survive in swamp forests. Shrub species, such as *Lespedeza bicolor*, dominated the shrub layer in areas with a high level of human disturbance at the forest edge. When the lower slope and the soil layer were thick, the shrubs were often unstratified owing to the high canopy density of the arbor layer. The richness of the herbaceous plants was high, resulting in the formation of *Herbage*, *L. gmelinii* (LH5) association. If the soil layer was thin, there were few tall shrubs, and *V. vitis‐idaea*, *L. gmelinii* (LV10) associations formed. The changes in the different association types were inconsistent. Our study found that the temperature type was the most important factor influencing *L. gmelinii*, followed by the soil type. Soil type considerably influences the distribution of LV10, which is related to the growth of such associations in brown taiga soils (Zhou, 1991).

In theory, only two datasets are needed to run the Maxent model. The first is the geographic distribution points displayed for the target species in the form of latitude and longitude. The second is the actual distribution area of the species and the environmental variables of the target area, which are predominantly climate data, terrain data, and soil data. Each association has its own specific habitat, which can be used for predicting the spatial geographic distribution of associations. We have the existence data for the distribution points and the environmental data associated with the distribution points, thus, meeting the two necessary conditions. Therefore, the Maxent model has applicability for association prediction. Regarding the environmental data selection, our study considered climate factors as dynamic variables and terrain and soil factors as static variables. However, the terrain and soil factors will also change under future climate scenarios (Richter & Markewitz, 2003). Moreover, subsequent research should consider data on biological factors to obtain more accurate prediction results.

Our study found that temperature was the most important factor affecting the distribution of *L. gmelinii* forests and most of its associations, and *L. gmelinii* forests are mainly distributed in the northern part of the Greater Khingan Range and in the Lesser Khingan Range. Yang et al. (2014) also demonstrated that temperature is the decisive factor for the potential distribution of *L. gmelinii* forests and that water conditions have a marginal limiting effect. These findings were consistent with the findings of previous studies (Li et al., 2006; Yang et al., 2014). However, future predictions are based on the relationship between the distribution of species and the environment, and because different models, environmental factors, and future climate scenarios, were considered, the prediction results of our study differed from those of previous studies (Chen, 2001; Li et al., 2006; Mu et al., 2021; Yang et al., 2014). For example, Li et al. (2006) studied the response of the spatial distribution of *L. gmelinii* to climate change from a statistical perspective by considering environmental variables, such as temperature, humidity, and precipitation, as factors, while in the present study, we classified different associations by humidity and then selected temperature and precipitation as factors. The terrain has a greater impact than the temperature on the distribution of some species. For example, Mu et al. (2021) reported that the order of importance of environmental factors is terrain, climate, soil, and elevation, which are the main factors affecting the distribution of *Larix principis*‐*rupprechtii* plantations. Although terrain cannot outweigh the influence of temperature in our study, the contribution of terrain can be greater than that of precipitation or soil in most *L. gmelinii* associations.

**FIGURE A5:**
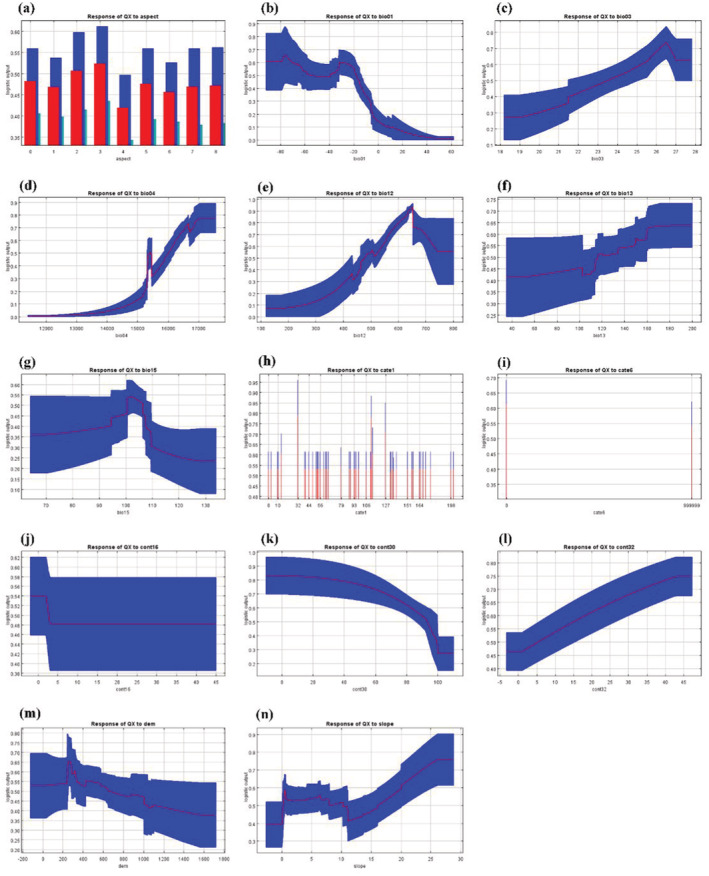
Variables Response curves for *Larix gmelinii* alliance. These curves show how each environmental variable affects the Maxent prediction. The curves show how the predicted probability of presence changes as each environmental variable is varied, while keeping all other environmental variables at their average sample value. The curves show the mean response of 10 replicate Maxent runs (red) and the mean ± one standard deviation (area in blue, the shade for categorical variables). (a) Aspect; (b) Bio01: Annual Mean Temperature (°C × 10); (c) bio03: Isothermality; (d) bio04: Temperature Seasonality; (e) bio12: Annual Precipitation (mm); (f) bio13: Precipitation of the Wettest Month (mm); (g) bio15: Precipitation Seasonality (%); (h) cate1: SU_SYM90 (Soil name in the FAO90 soil classification system); (i) cate6: SWR (soil moisture content); (j) cont16:T_CACO3: Real (Surface carbonate or lime content) (%weight); (k) cont30: S_BS: Real (Basic saturation of the bottom layer) (%); (l) cont32: S_CACO3: Real (Bottom carbonate or lime content) (%weight); (m) dem: Altitude(m); (n) slope (°). For specific explanations of environmental factors, please refer to Table 2.

**FIGURE A6:**
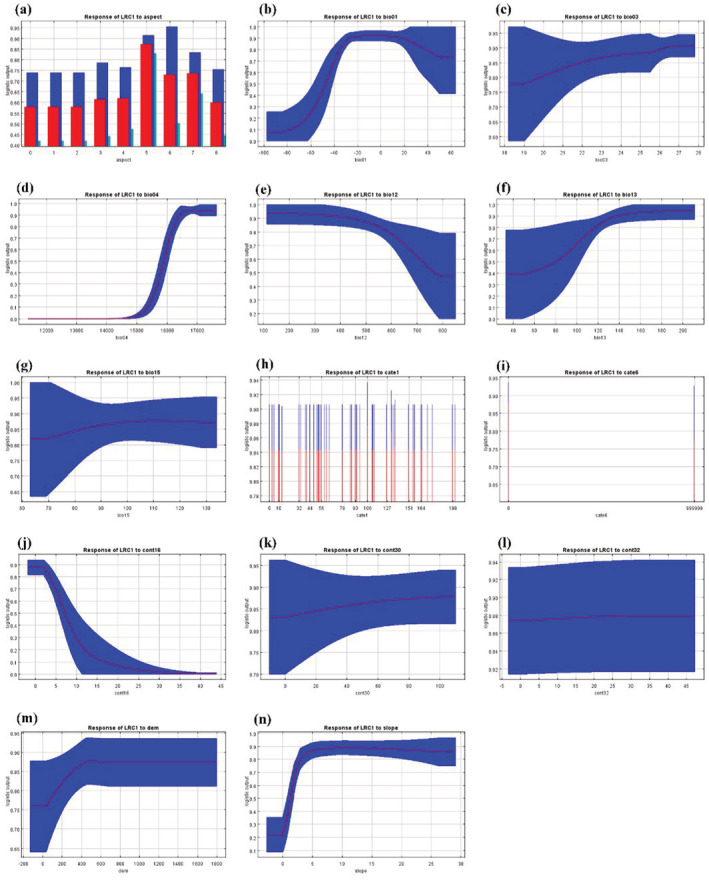
Variables Response curves for LRC1. For specific explanations of environmental factors, please refer to Table 2

**FIGURE A7:**
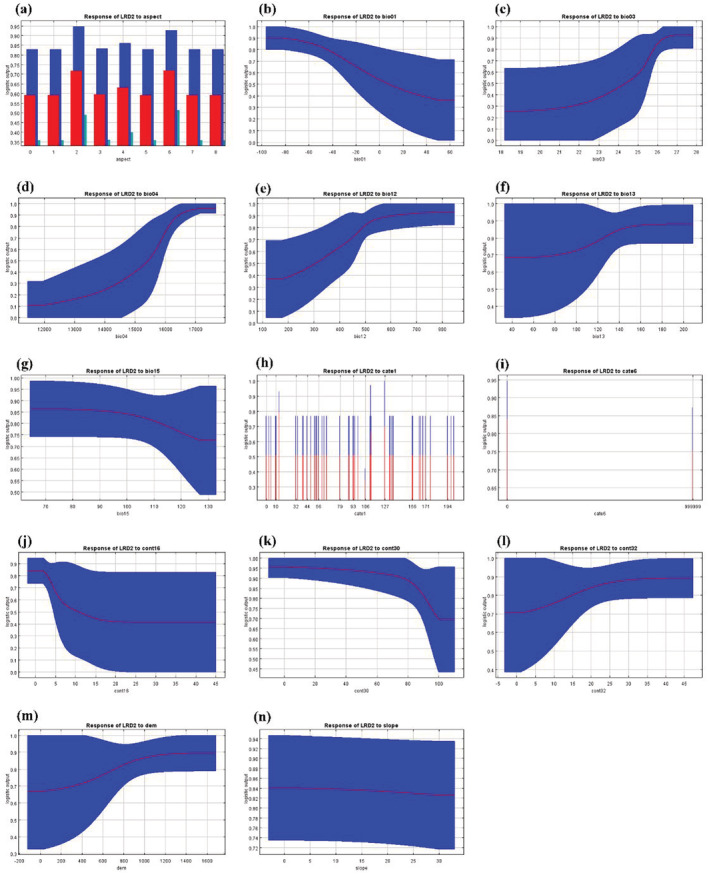
Variables Response curves for LRD2. For specific explanations of environmental factors, please refer to Table 2

**FIGURE A8:**
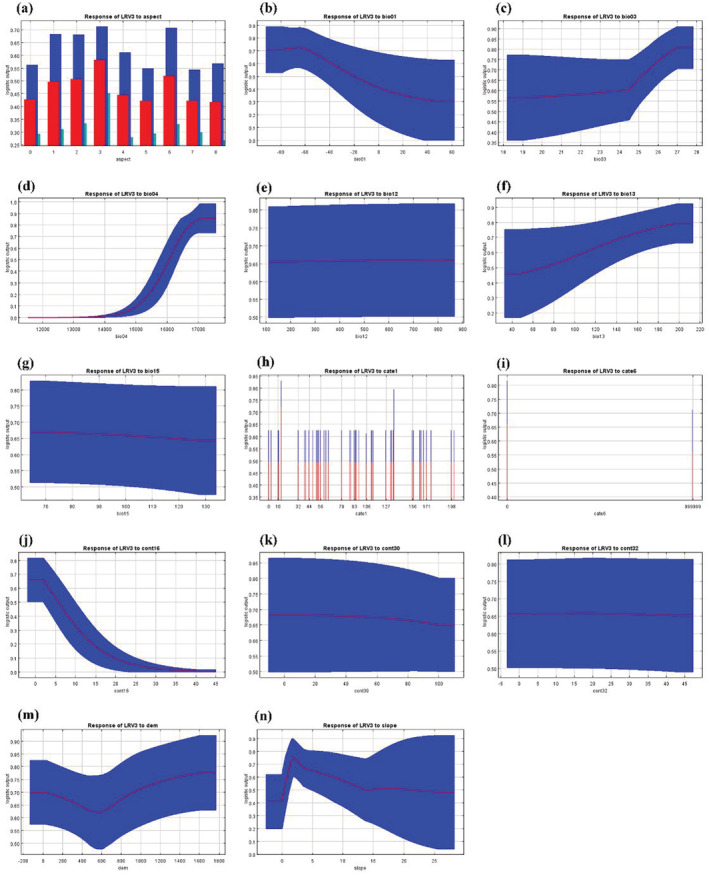
Variables Response curves for LRV3. For specific explanations of environmental factors, please refer to Table 2

**FIGURE A9:**
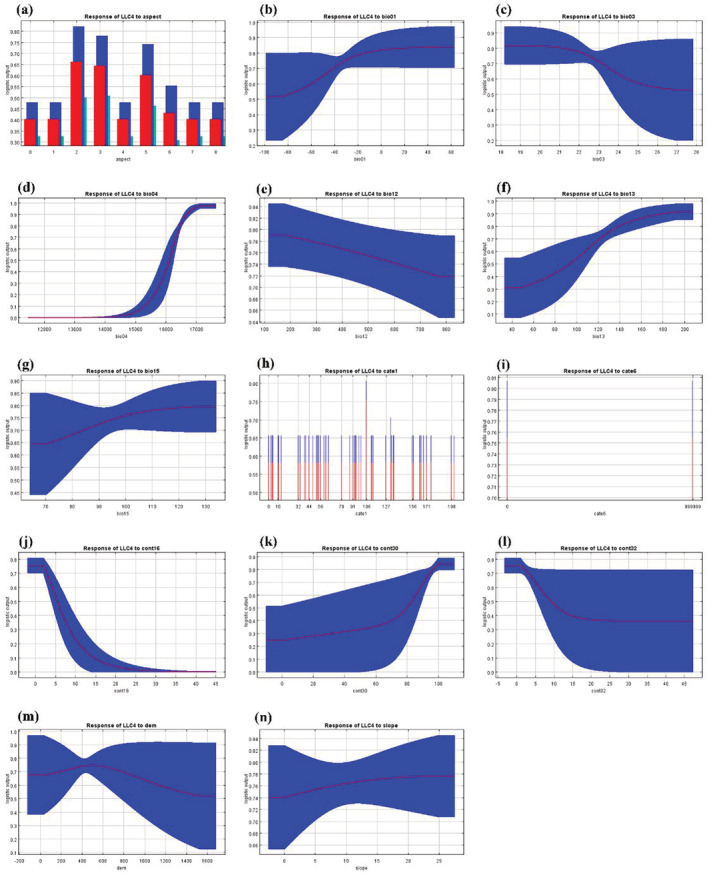
Variables Response curves for LLC4. For specific explanations of environmental factors, please refer to Table 2

**FIGURE A10:**
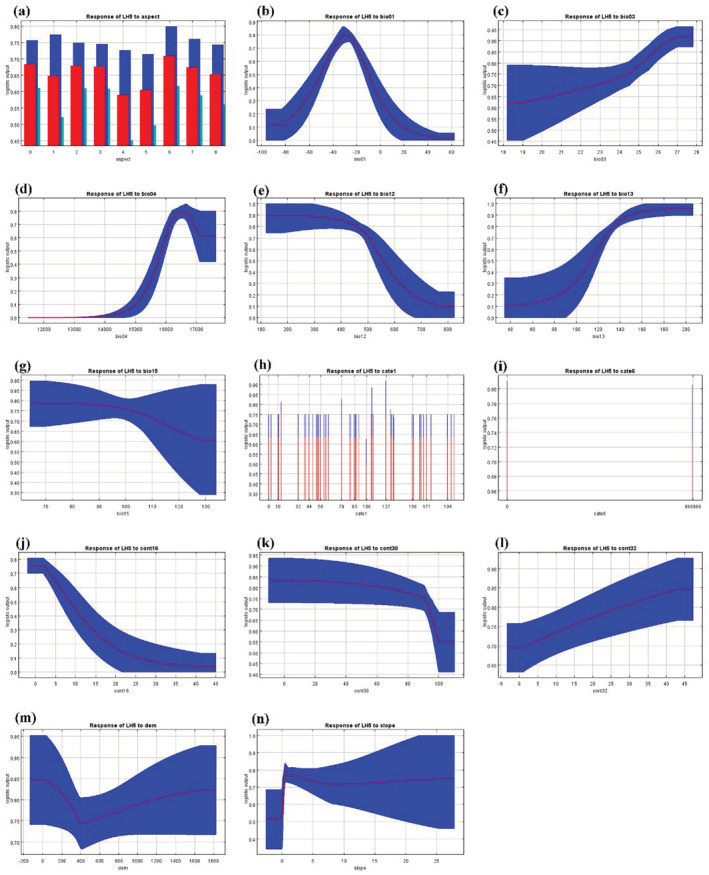
Variables Response curves for LH5. For specific explanations of environmental factors, please refer to Table 2

**FIGURE A11:**
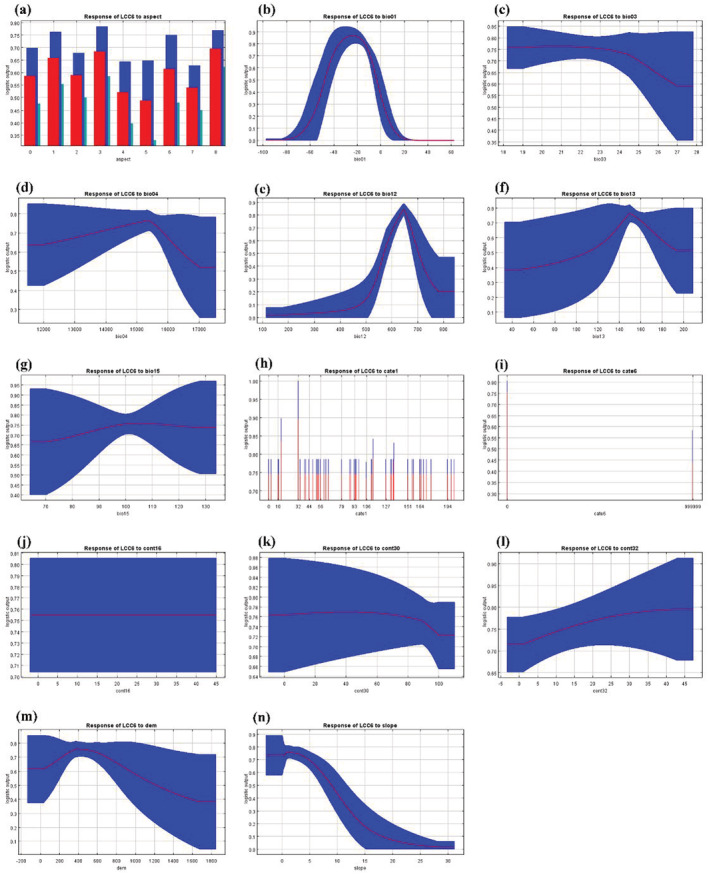
Variables Response curves for LCC6. For specific explanations of environmental factors, please refer to Table 2

**FIGURE A12:**
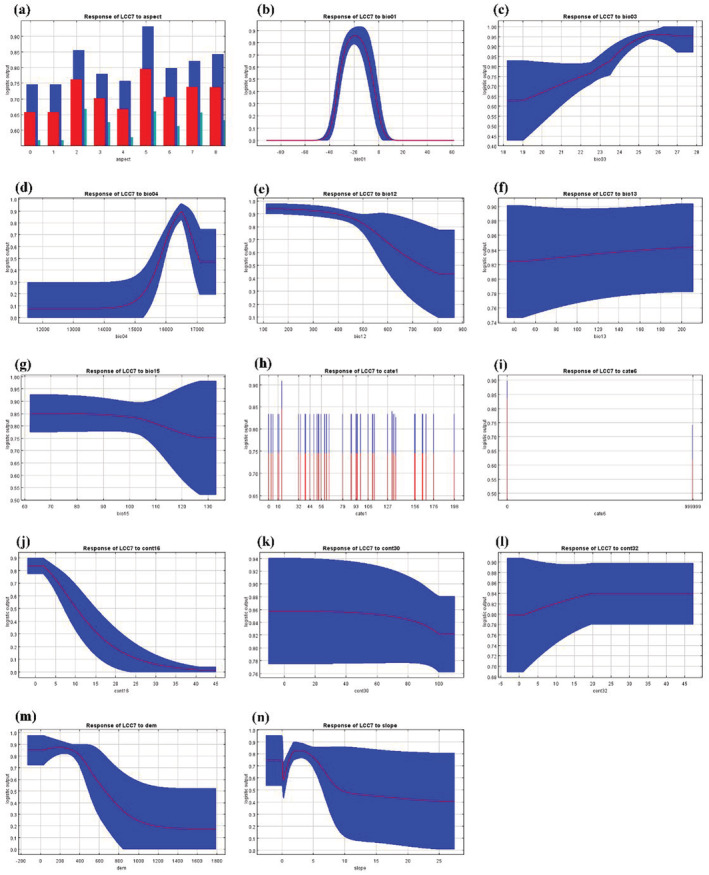
Variables Response curves for LCC7. For specific explanations of environmental factors, please refer to Table 2

**FIGURE A13:**
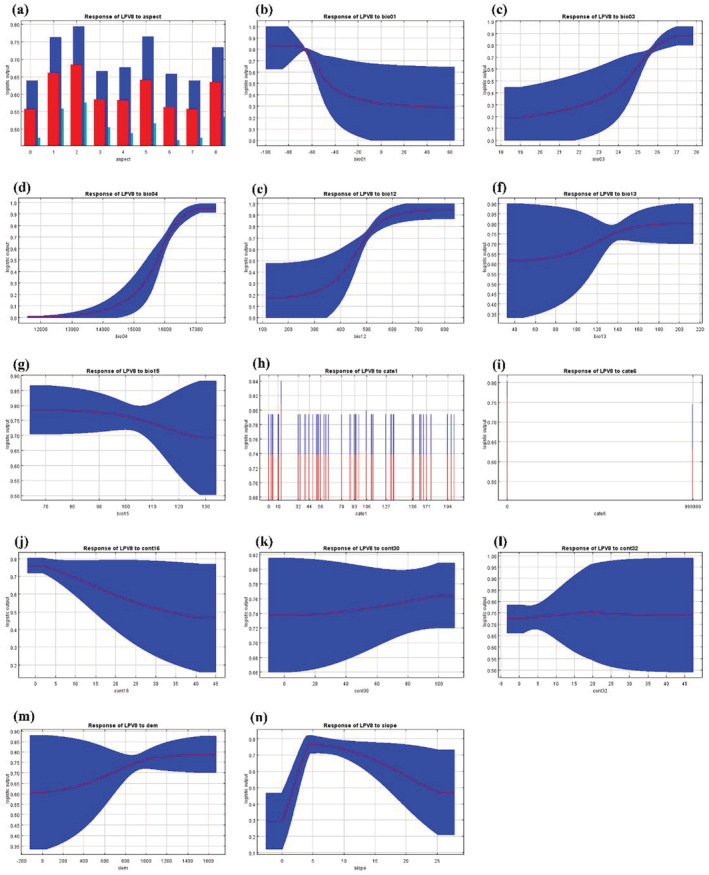
Variables Response curves for LPV8. For specific explanations of environmental factors, please refer to Table 2

**FIGURE A14:**
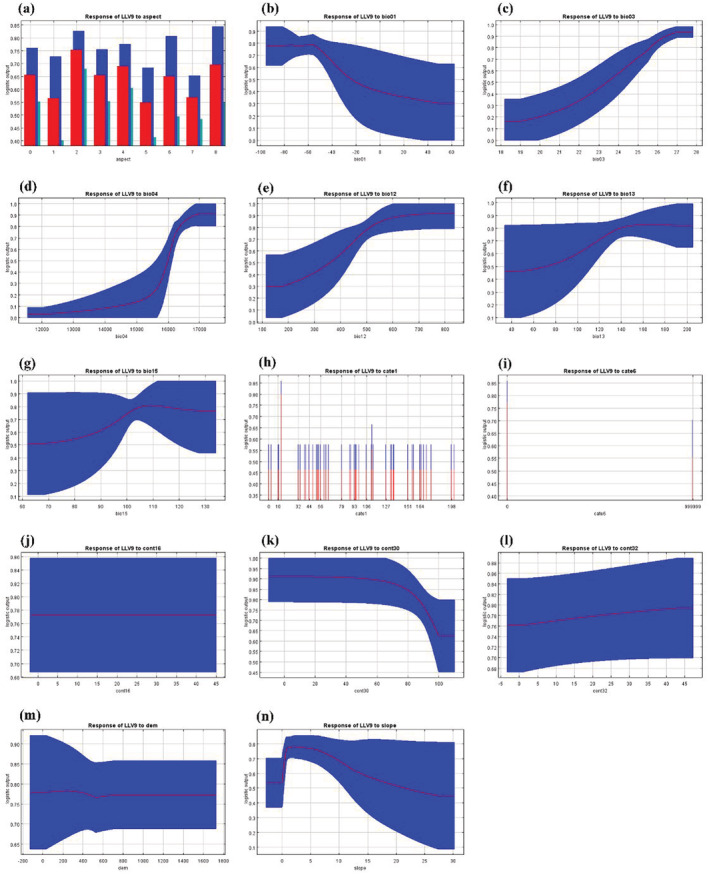
Variables Response curves for LLV9. For specific explanations of environmental factors, please refer to Table 2

**FIGURE A15:**
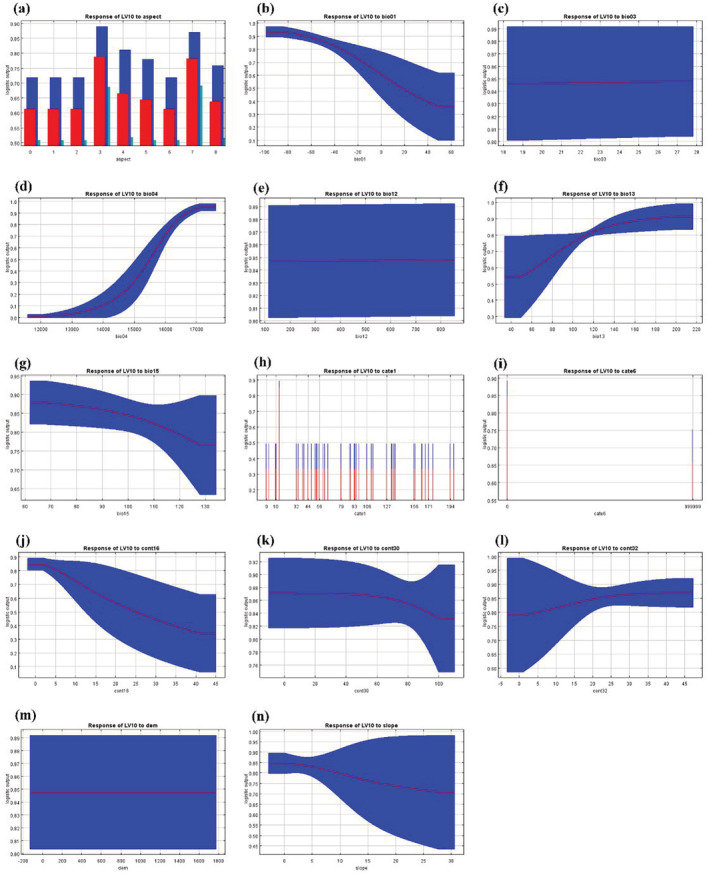
Variables Response curves for LV10. For specific explanations of environmental factors, please refer to Table 2

**FIGURE A16:**
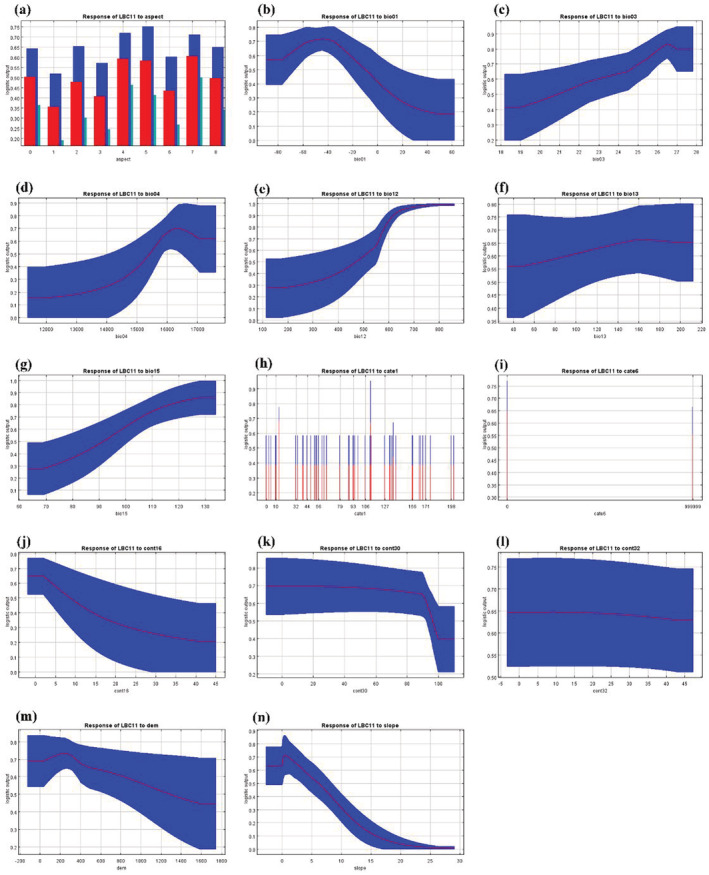
Variables Response curves for LBC11. For specific explanations of environmental factors, please refer to Table 2

**FIGURE A17:**
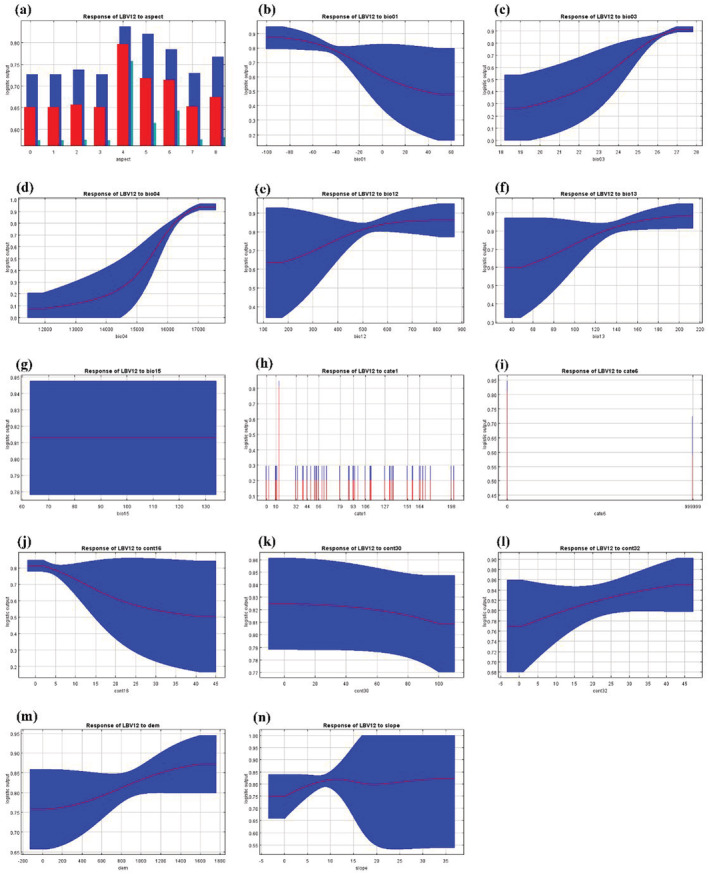
Variables Response curves for LBV12. For specific explanations of environmental factors, please refer to Table 2

**FIGURE A18:**
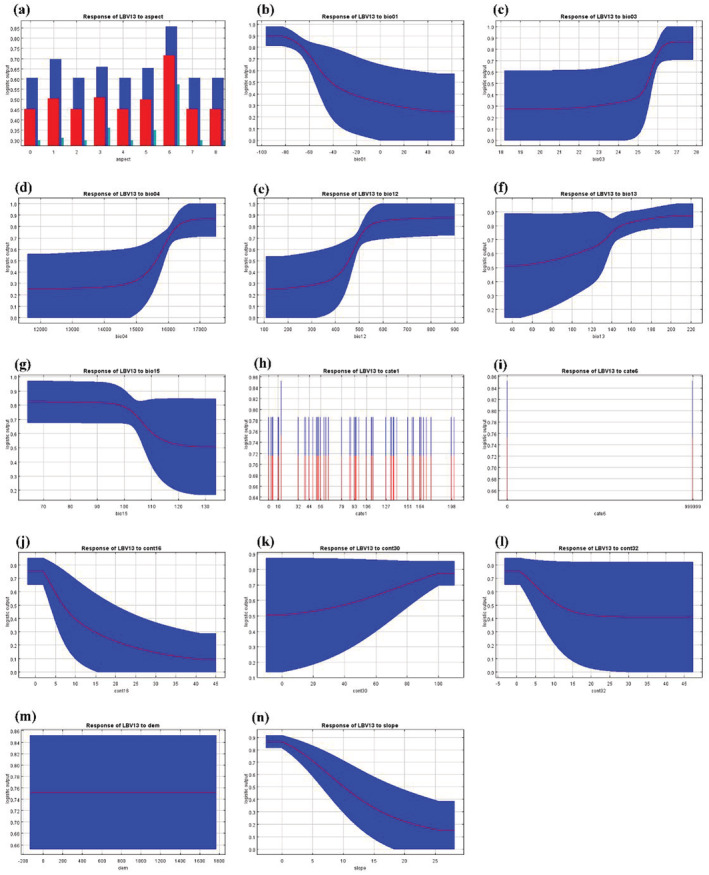
Variables Response curves for LBV13. For specific explanations of environmental factors, please refer to Table 2

The response curves of *L. gmelinii* alliance and associations showed how each environmental variable affects the Maxent prediction (Figures A5 and A6, A7, A8, A9, A10, A11, A12, A13, A14, A15, A16, A17, A18 in Appendix 1). The curves showed how the predicted probability of presence changes as each environmental variable was varied, keeping all other environmental variables at their average sample value. With the increase in the average annual temperature (bio01), the probability of the existence of *L. gmelinii* alliance suitable habitats will decrease. We also analyzed the response of the *L. gmelinii* alliance and associations with the suitable area increasing or decreasing due to climate change under different climate scenarios (Figures 5 and 6). We chose the point of the *L. gmelinii* alliance as the point for the study of the change in the suitable area so that the study location can meet the basic conditions for the growth of *L. gmelinii*. It is possible to determine the change in the suitable area by using the numerical difference between the distribution under different climate scenarios in the future and the current distribution. This analysis can selectively analyze the environmental factors for the increase of the suitable area according to the change or reduced impact. Increasing temperature, precipitation increase, and precipitation seasonal dispersion are the main causes for the reduction in the suitable area for the *Larix gmelinii* alliance.

In terms of clump physiology, *L. gmelinii* has strong drought resistance and can grow under mild drought conditions (Sugimoto et al., 2002). Under climate warming, the distribution of plant species tends to shift to habitats at high latitudes or altitudes (He et al., 2019). In the future, suitable areas for each association are expected to shrink by varying degrees. Using a logistic regression model, Leng et al. (2006) predicted that *L. gmelinii* would retreat 200 km northward by 2050 and 300 km northward by 2100. LRC1, LH5 and LCC6 showed a pronounced northward shift trend under future climate change simulations, which was in line with the results of previous studies (Chen, 2000; He et al., 2019; Yang et al., 2014). Although the area expansion is relatively small, associations shift during the transition of the area, which requires further attention. Under such circumstances, reducing human disturbance is recommended. The sensitivity of some species to climate change may be overlooked when the range of observations is limited to *L. gmelinii*. When the general living conditions support multiple species with similar functions, or some species contribute less to the general living conditions, or when the characteristics are controlled mainly by the abiotic environment, the characteristics of the ecosystem will be insensitive to species loss (Hooper et al., 2005).

For different *L. gmelinii* associations, different management measures are required for each association because of different habitat conditions, composition structure, growth, development, and renewal succession trends (Estrada Valdés et al., 2021; González de Andrés et al., 2018; Jia et al., 2016). Areas with good site conditions should be selected for performing thinning, and the forest spatial structure should be adjusted and optimized through thinning (Zhang et al., 2018). The mesogenic drought habitat associations have the characteristics of a dry and cold climate and mainly contain leafy plants but no big leafy plants (Zhou, 1991). Considering the *R. davuricum*, *L. gmelinii* (LRC1, LRD2, and LRV3) association, most of these forests are in the overripening stage, and there are often diseases that affect the development of trees, but understory *L. gmelinii* saplings can form a multi‐layer heterogeneous forest (Zhou, 1991). According to the prediction of future climate scenarios, the amplification area of LRC1 is large, which indirectly reflects that the dominant position of *C. callitrichos* is relatively strong in future environmental adaptability. In the future, we should pay attention to the pest control of the *R. davuricum*, *L. gmelinii* associations, and pay attention to the growth and renewal progress of larch saplings. The *C. callitrichos*, *L. bicolor*, *L. gmelinii* (LLC4) association is mainly distributed on the northern edge of the study area. Compared with the *R. davuricum*, *L. gmelinii* associations, the number of this group is relatively small. It is necessary to strengthen the work of tending young forests and combine harvesting and cultivation. Layering phenomenon and lamellar structure of the *Herbage*, *L. gmelinii* association (LH5) are simple, and this kind of *L. gmelinii* forest is the same age forest (Zhou, 1991). Although LH5 is more common, in the future it is also necessary to focus on protecting the reduced areas of LH5 suitable areas, implementing artificial promotion updates, and increasing protection and attention. The natural regeneration of LCC6 *L. gmelinii* forest is poor, but the trees grow lush and accumulate large amount; this is a forest type with high economic value in the northern part of Xiao Hinggan Ling (Zhou, 1994). The suitable area of LCC6 and LCC7 under the prediction of future climate scenarios changes greatly, and the protection of wild resource plants in the area should be strengthened when using forest grassland for the production of related economic sideline industries. Furthermore, the recovery of forests after a fire is generally due to undamaged or slightly damaged trees (Oreshkova et al., 2013). Therefore, it is necessary to increase the number of LPV8 samples that face the most serious fire loss (Chen et al., 2015; Makoto et al., 2011). LPV8 is also critical as an important net resource for maintaining the economic development of the community. LLV9 Natural regeneration of *L. gmelinii* is poor due to the shade and wet forest, thick moss and lichen (Zhou, 1991). For LLV9, attention should be paid to the combination of artificial and natural regeneration, and the growth of larch should be promoted by rational utilization of moss and lichen. Understory natural regeneration of LV10 is good, and *Vaccinium* is widespread under such *L. gmelinii* with a frequency of 100%, and the soil of this kind of association is moist and has good drainage (Zhou, 1991). In the future, we should pay attention to water conservation and minimize soil erosion. The suitable area of LBC11 is the largest in our study, and the adaptation of this association to climate change is relatively good in future climate scenarios. In the future, we should pay attention to the impact of human factors on this cluster and protect the existing habitats as far as possible. Previous studies have demonstrated the effects of fixed conditions on plants and animal communities, such as the long‐term absence of rain (Vicente‐Serrano et al., 2020). As far as possible, continuous conditions should be artificially created for climate‐sensitive association types, such as creating wet and moist conditions for wet association groups (LBV12 and LBV13).

## 5 CONCLUSIONS

The responses of different *L. gmelinii* association types to climate change showed a divergent trend. Temperature is the most important factor affecting the distribution of *L. gmelinii* forests and their associations under different climate scenarios. Compared with the contribution of the variables analyzed in the Maxent model, some variables such as precipitation with a relatively low contribution also play a role in causing the increase or decrease of suitable area. Future studies will consider both different species and focus on conserving *L. gmelinii* after relocation and its associated economic species to sustain different forest ecosystems and their associations under the backdrop of global climate warming.

## AUTHOR CONTRIBUTIONS

**Jing‐hua Yu**: Conceptualization (lead); Funding Acquisition (lead); Project administration (lead); Supervision (lead); Investigation (equal); Writing–reviewing and editing (equal). **Chen Chen**: writing–original draft (lead); Formal analysis (supporting); Visualization (lead); Writing–reviewing & editing (equal). **Xi‐juan Zhang**: writing–original draft (supporting); Formal analysis (lead); Writing–reviewing and editing (equal). **Ji‐zhong Wan**: Supervision (supporting); Writing–reviewing and editing (equal). **Fei‐fei Gao**: Formal analysis (supporting); Writing–reviewing and editing (equal). **Shu‐sheng Yuan**: Investigation (equal); Writing–reviewing and editing (equal). **Tian‐tian Sun**: Writing–reviewing and editing (equal). **Zhen‐dong Ni**: Data curation (supporting).

## CONFLICT OF INTEREST

The authors declare that they have no known competing financial interests or personal relationships that could have influenced the work reported in this study.

### OPEN RESEARCH BADGES

This article has earned Open Data, Open Materials and Preregistered Research Design badges. Data, materials and the preregistered design and analysis plan are available at https://doi.org/10.6084/m9.figshare.20438595.

## Data Availability

The data of this paper is stored in https://doi.org/10.6084/m9.figshare.20438595.

## APPENDIX 1 Introduction of association types

LRC1 (Ass. *Carex callitrichos*, *Rhododendron davuricum*, and *Larix gmelinii*) was present in the cold temperate coniferous forest area on the northeast and southeast slopes (1–28°) of the Huzhong National Nature Reserve, Duobukur National Nature Reserve, Nanwenghe National Nature Reserve, and Chuonahe National Nature Reserve. Such low and medium degree slopes are common at approximately 400–800 m. The main forest layer consisted of *L. gmelinii*, *Betula platyphylla*, and *Quercus mongolica*. The shrub layer was dominated by *R. davuricum* and *Vaccinium vitis‐idaea*, and the herb layer was dominated by ferns and *C. callitrichos*.

LRD2 (Ass. *Deyeuxia pyramidalis*, *R. davuricum*, and *L. gmelinii*) was present in the cold temperate coniferous forest area on the southeast, northeast, and middle–low slopes of the Huzhong National Nature Reserve and Da Hinggan Ling Hanma National Nature Reserve, at an altitude of approximately 500–1000 m. The main forest layer was dominated by *L. gmelinii* and included a sparse population of *B. platyphylla*. The irrigated layer was dominated by *R. dauricum* and accompanied by a small population of *Spiraea salicifolia*. *Deyeuxia arundinacea* dominated the herb layer, accompanied by non‐layered *V. vitis‐idea* and *Ledum palustre*, and the lower layer included *Rosa davurica*, *Sorbaria sorbifolia*, *Pyrola asarifolia* subsp*. incarnata*, *Ribes janczewskii*, *Convallaria majalis* Linnaeus, *Maianthemum bifolium*, and *Deyeuxia purpurea*.

LRV3 (Ass. *V. vitis‐idea*, *R. davuricum*, and *L. gmelinii*) was found on the lower or middle regions of the southeast slopes of Genhe, Inner Mongolia, at an altitude of approximately 890–1100 m. The main forest layer consisted of *L. gmelinii*, with a small population of *B. platyphylla*. The shrub layer was dominated by *Rhododendron dauricum* and accompanied by a small population of *Pinus pumila*. The herb layer included *L. palustre* and *V. vitis‐idea*, accompanied by a sparse populations of *D. purpurea*, *Vicia pseudo‐orobus*, *Sanguisorba officinalis*, *Iris uniflora*, *Peucedanum terebinthaceum*, and *Juniperus davurica*.

LLC4 (Ass. *C. callitrichos*, *Lespedeza bicolor*, and *L. gmelinii*) was found on the northeast and southwest slopes and was located in the middle or lower parts of the slopes of the Chuonahe National Nature Reserve, at an altitude of approximately 400–600 m. The forest layer mainly consisted of *Q. mongolica* and *B. platyphylla*. The shrub layer was mainly populated by *L. bicolor*, accompanied by sparse populations of *R. davuricum* and *Vaccinium vitisidea*. The herb layer was dominated by *C. callitrichos*.

LH5 (Ass. *Herbage* and *L. gmelinii*) was mainly distributed on the sun and semi‐sun slopes of the Ergun National Nature Reserve and Da Hinggan Ling Hanma National Nature Reserve of the cold temperate coniferous forest subzone. The slope is generally 2–10°. Most of these associations are derived from the forests of *Q. mongolica* and *Larix olgensis*. The main forest layer consisted of *L. gmelinii*, and the shrub layer included *Spiraea media*, *Vaccinium uliginosum* Linn., *Sorbaria sorbifolia*, and *Philadelphus schrenkii*. The herb layer mainly included *D. purpurea*, *Carex lanceolata*, *Carex ussuriensis*, *D. pyramidalis*, and *Pyrola rotundifolia*.

LCC6 (Ass. *C. lanceolata*, *Corylus mandshurica*, and *L. gmelinii*) was derived from broad‐leaved Korean pine forests of the Youhao National Nature Reserve and was distributed in terraces or second‐level terraces at an altitude of 300–500 m. *L. gmelinii* was the dominant tree species, the main shrubs were *C. mandshurica* and *Aralia elata*, and the main herbs were *Bolboschoenus yagara* and *Filipendula palmata*.

LCC7 (Ass. *C. callitrichos*, *Corylus heterophylla*, and *L. gmelinii*) was found in the middle or lower parts of the northeast and northwest slopes of the Duobukur National Nature Reserve, Nanwenghe National Nature Reserve, and Chuonahe National Nature Reserve, at approximately 200–500 m a.s.l. The trees primarily included *B. platyphylla*, *Populus davidiana*, and *L. gmelinii*. The shrub layer was mainly composed of *C. heterophylla* Fisch. The herb layer included *C. callitrichos* and was accompanied by sparse populations of *D. purpurea*, *F. palmata*, *Cimicifuga dahurica*, *S. officinalis*, and some ferns.

LPV8 (Ass. *V. vitis‐idaea*, *Pinus pumila*, and *Larix gmelinii*) was the zonal vegetation of the cold temperate coniferous forest belt in the mountainous area of the Daxinganling Mountains, which was the highest altitude distribution of the *L. gmelinii* forest. LPV8 was mainly distributed at the mountain top, on the ridge, on the upper part of the slope, and on the broad watershed. The habitat temperature was low, the wind was high, and tree growth was affected. Only thick pines occupied the shrub layer. *Larix gmelinii* was the dominant species in the forest layer, and a small population of *B. platyphylla* was also present in the main forest layer. The shrub layer was dominated by *P. pumila*, with sparse populations of *Alnus mandshurica*, *Sorbus pohuashanensis*, and *R. dauricum*. *Vaccinium vitis‐idaea* dominated the shrub layer, and *L. palustre* and *R. davurica* were randomly distributed in the shrub layer.

LLV9 (Ass. *V. vitis‐idaea*, *L. palustre*, and *L. gmelinii*) was sporadically distributed in the cold temperate coniferous forest subzone in the middle or lower part of the mountain area. It was mostly distributed in floodplains and terraced riverbank valleys, and the slope was gentle, mostly within 5° (Zhou, 1991). The tree layer mainly consisted of *L. gmelinii* as a mature, pure forest with an occasional occurrence of *B. platyphylla*. The shrub layer was dominated by *L. palustre* and *D. arundinacea*, and accompanied by sparse populations of *R. davurica*, *V. uliginosum* Linn., *V. vitis‐idaea* Linn., *Betula middendorfii*, and *Equisetum pratense* Ehrh. The moss layer was extremely underdeveloped.

LV10 (Ass. *V. vitis‐idaea* and *L. gmelinii*) represented cold and humid habitat conditions and was marginally distributed in the Daxinganling Mountains. It was concentrated in the subzone of the cold temperate coniferous forest in the middle of the mountain. The soil was the brown taiga forest soil and was moist and well‐drained (Zhou, 1991). Leaves of deciduous conifers, consisting of *L. gmelinii*, were dominant among standing trees and sometimes mixed with populations of *B. platyphylla* and *Pinus sylvestris* var. *mongolica*. The evergreen shoots *of V. vitis‐idaea* Linn. were the dominant layers in the shrub layer. *Carex lanceolata* Boott, *D. pyramidalis*, *F. palmata*, and *D. purpurea* were common in the shrub layer.

LBC11 (Ass. *Carex schmidtii*, *Betula fruticosa*, and *L. gmelinii*) was generally located in flat bottoms, valleys, and low‐lying areas. The soil in this region was gleyed brown coniferous forest or swamp soil. The permafrost layer was deep and belonged to a continuous frozen soil area. This type of vegetation was characterized by distinct vegetation stratification. *Larix gmelinii* was the dominant species in the tree layer and was sometimes accompanied by *B. platyphylla*. The shrub layer was typically dominated by *B. fruticosa*. The second shrub layer was composed of *V. uliginosum* and *L. palustre*. The herb layer was mainly composed of *C. schmidtii*, and accompanied by sparse populations of *D. pyramidalis*, *F. palmata*, *Equisetum sylvaticum* L., *D. purpurea*, *V. uliginosum*, *Saussurea neoserrata*, *V. vitis‐idaea* Linn, *P. asarifolia* subsp*. incarnata*, *R. davurica*, and *S. officinalis*.

LBV12 (Ass. *V. vitis‐idaea*, *B. fruticosa*, and *L. gmelinii*) was located in plains, valleys, low‐lying areas, and slope feet, at an elevation of 200–1000 m. The tree layer was mainly composed of *L. gmelinii*. The shrub layer was mainly composed of *B. fruticosa*, and accompanied by sparse populations of *L. palustre*, *D. pyramidalis*, *R. davurica*, *V. uliginosum*, and *Chamerion angustifolium*. The grass layer was mainly composed of *V. vitis‐idaea* Linn., accompanied by sparse populations of *Ribes mandshuricum* and *Maianthemum trifolium*.

LBV13 (Ass. *V. vitis‐idaea*, *B. middendorfii*, and *L. gmelinii*) was located on flat, middle, or lower slopes at an elevation of 800–1000 m. The main forest layer was dominated by *L. gmelinii*. The first shrub layer was dominated by *B. middendorfii* and occasionally by *B. fruticosa*. The second shrub layer was dominated by *L. palustre* and occasionally by *R. davurica*, *C. angustifolium*, *L. gmelinii*, *Carex karoi*, and *D. pyramidalis*. The herb layer was dominated by *V. vitis‐idaea* Linn., and the moss layer was not well‐developed.

**TABLE A1:** Correlation matrix of environmental factors

^a^	bio01	bio03	bio04	bio12	bio13	bio15	dem	slope	aspect	cate1	cate6	cont16	cont30	cont32
bio01	1													
bio03	0.657473	1												
bio04	0.733163	0.213426	1											
bio12	0.186838	0.150716	0.253728	1										
bio13	0.149581	0.033444	0.236126	0.79839	1									
bio15	0.156788	0.291245	0.175538	0.687054	0.1496651									
dem	0.777358	0.691907	0.249341	0.285616	0.174428	0.30289	1							
slope	0.395976	0.441496	0.002272	0.208309	0.205082	0.06742	0.501642	1						
aspect	0.002592	0.005105	0.010064	0.016003	0.001717	0.021663	0.026513	0.017422	1					
cate1	0.171949	0.087765	0.117635	0.133557	0.169848	0.01606	0.159395	0.019313	0.007772	1				
cate6	0.055019	0.044463	0.0814	0.091312	0.072954	0.083413	0.025751	0.057263	0.003298	0.214457	1			
cont16	0.164292	0.17712	0.019047	0.360645	0.223044	0.299146	0.045453	0.2189	0.01417	0.223916	0.030941	1		
cont30	0.042512	0.001853	0.02787	0.107215	0.063749	0.082173	0.079851	0.004965	0.002577	0.130441	0.85703	0.242455	1	
cont32	0.414123	0.375421	0.173046	0.026459	0.070075	0.010309	0.442251	0.328522	1.54E‐05	0.083864	0.077412	0.206713	0.13659	1

^a^ Please refer to the Table 2 for a detailed introduction to environmental factors.
